# Driving β_2_- While Suppressing α-Adrenergic Receptor Activity Suppresses Joint Pathology in Inflammatory Arthritis

**DOI:** 10.3389/fimmu.2021.628065

**Published:** 2021-06-17

**Authors:** Denise L. Bellinger, Carlo Wood, Jon E. Wergedal, Dianne Lorton

**Affiliations:** ^1^ Department of Human Anatomy and Pathology, Loma Linda University School of Medicine, Loma Linda, CA, United States; ^2^ Musculoskeletal Disease Center, VA Loma Linda Healthcare System, Loma Linda, CA, United States; ^3^ Departments of Medicine and Biochemistry, Loma Linda University, Loma Linda, CA, United States; ^4^ Hoover Arthritis Research Center, Banner Health Research Institute, Sun City, AZ, United States

**Keywords:** terbutaline, sympathetic nerves, bone and cartilage sparing, disease severity, adrenergic signaling, rheumatoid arthritis, bone marrow adiposity, phentolamine

## Abstract

**Objective:**

Hypersympathetic activity is prominent in rheumatoid arthritis, and major life stressors precede onset in ~80% of patients. These findings and others support a link between stress, the sympathetic nervous system and disease onset and progression. Here, we extend previous research by evaluating how selective peripherally acting α/β_2_-adrenergic drugs affect joint destruction in adjuvant-induced arthritis.

**Methods:**

Complete Freund’s adjuvant induced inflammatory arthritis in male Lewis rats. Controls received no treatment. Arthritic rats then received vehicle or twice-daily treatment with the α-adrenergic antagonist, phentolamine (0.5 mg/day) and the β_2_-adrenergic agonist, terbutaline (1200 µg/day, collectively named SH1293) from day (D) of disease onset (D12) through acute (D21) and severe disease (D28). Disease progression was assessed in the hind limbs using dorsoplantar widths, X-ray analysis, micro-computed tomography, and routine histology on D14, D21, and D28 post-immunization.

**Results:**

On D21, SH1293 significantly attenuated arthritis in the hind limbs, based on reduced lymphocytic infiltration, preservation of cartilage, and bone volume. Pannus formation and sympathetic nerve loss were not affected by SH1293. Bone area and osteoclast number revealed high- and low-treatment-responding groups. In high-responding rats, treatment with SH1293 significantly preserved bone area and decreased osteoclast number, data that correlated with drug-mediated joint preservation. SH1293 suppressed abnormal bone formation based on reduced production of osteophytes. On D28, the arthritic sparing effects of SH1293 on lymphocytic infiltration, cartilage and bone sparing were maintained at the expense of bone marrow adipocity. However, sympathetic nerves were retracted from the talocrural joint.

**Conclusion and Significance:**

Our findings support a significant delay in early arthritis progression by treatment with SH1293. Targeting sympathetic neurotransmission may provide a strategy to slow disease progression.

## Introduction

Rheumatoid arthritis (RA) afflicts about 1% of adults worldwide. RA is a debilitating chronic inflammatory autoimmune disease of the joints, with no cure and no strategy for prevention (1). In RA, inflammation, immune cell infiltration into the synovium of affected joints and circulating proinflammatory cytokines are characteristic features of disease. Although, the etiology of RA remains obscure, recent advances in our understanding of the underlying disease pathophysiology have driven the development of novel efficacious anti-rheumatic therapies. Still, many RA patients are unresponsive or have side effects to current treatments, necessitating additional novel treatments for RA.

One potential target for RA treatment may be the autonomic nervous system. Both articular and extra-articular manifestations of RA are under the influence of the central and peripheral autonomic activity in two ways (2, 3). First, direct sympathetic innervation of the affected joints modulates disease activity by norepinephrine activating α- and/or β-adrenergic receptors (ARs) expressed in bone, cartilage and the vasculature (4, 5). Secondly, sympathetic nerves also supply immune organs that can indirectly affect joint destruction by directing the immune response to arthritogenic antigens. Interestingly, both the distribution and density of sympathetic nerves in immune organs dynamically changes in response to disease induction (6). Via these direct and indirect routes, the sympathetic nervous system (SNS) exerts direct and indirect regulation of joint physiology. A better understanding of how these routes of sympathetic regulation impact joint integrity and pathology may provide a unique target for treating localized joint pathology, as well as modulating disease causing immune cells and secondary systemic effects of RA.

The SNS monitors and influences homeostasis of articular joints *via* noradrenergic nerves distributing to the various tissue components that comprise them. Sympathetic regulation of joint integrity is complex because of its diverse cellular targets and the selective expression of AR subtypes these targets express. Norepinephrine released from sympathetic nerves in response to a stimulus activates α- and/or β-ARs expressed in target tissues in immune organs and articular joints. The effects of norepinephrine on target tissues depend on the type(s) of cell surface receptor(s) the target cells express, and the local norepinephrine concentration at the effector junction. Norepinephrine acts postsynaptically on β_2_- or α_1_-ARs, but also presynaptically on α_2_-ARs to inhibit its release (7). The α_1_-ARs are often greater expressed than β_2_-ARs in target tissues, and catecholamines have a higher affinity for α-ARs relative to β_2_-ARs. High norepinephrine concentrations (e.g., 100 µmol/L) activate both α- and β-ARs, whereas concentrations below this range activate α-ARs (8, 9). Additionally, α_2_-ARs expressed in the limbic system, hypothalamus and brainstem suppress sympathetic outflow (10–15). Hypersympathetic activity in RA and adjuvant-induced arthritis may disrupt normal homeostatic regulation *via* changes in adrenergic receptor activity in affected joints. The SNS also monitors and influences immune homeostasis *via* nerves that innervate both primary and secondary immune organs (16, 17). The distribution and density of these nerves is dynamic, changing in response to changes in their microenvironment. These changes include immune activation and cytokine secretion, indicating that the immune system can influence the SNS, which in turn directly influences immune cells in their microenvironment. Lymphocytes that mediate adaptive immunity predominantly express β_2_-ARs, while cells of innate immunity, like macrophages, express β_2_, α_1_- and α_2_-ARs, typically with β_2_-ARs driving anti-inflammatory and α-ARs promoting proinflammatory macrophage activity (18). Thus, both nerve density, proximity of nerves to target cells and activation of AR subtype(s) in real time determines the robustness of the immune response to an arthritis-inducing challenge, and the effector response in joint tissues.

In the adjuvant-induced rat model of RA, we were the first to report that α- and β-adrenergic drugs administered during different phases of disease exert dynamic time-dependent and receptor-specific changes in immune response profiles that determine disease severity (19). Treatment with either an α-antagonist or a β_2_-agonist from disease induction through disease onset exacerbated disease. Conversely, treatment with an α-antagonist or a β_2_-agonist from disease onset through severe disease (late phase) reduced disease severity (19). Unique cytokine profiles from splenic and hind limb-draining lymph nodes (DLNs) mediated improved or worsened disease outcomes depending on (1) the selective adrenergic treatment, and (2) the disease phase of drug administration. Other researchers later confirmed our findings in a murine model of experimental arthritis (20–22). Collectively, we established that the SNS has a dual immunoregulatory role in inflammatory arthritis (IA) by driving pro- and anti-inflammatory effects in early and late phases of disease, respectively. In a follow-up study (23), we determined that normal β_2_-AR signaling of the immune response to challenge with complete Freund’s adjuvant (CFA) is derailed in spleen cells and that β_2_-ARs in DLN cells switch their β_2_-AR signaling from cAMP-protein kinase A (PKA) to a mitogen activated protein kinase (MAPK) pathway (17). Changes in immune organ signaling prevent sympathetic regulation of immune cells *via* β_2_-ARs in the spleen. Moreover, after established disease they promote autoreactive immune cell development in DLNs. These findings indicate a complicated immunoregulatory role for the SNS in the development and progression of IA (2, 19–24). Moreover, they provide the rationale for our more recent strategy of therapeutically targeting the SNS *via* brainstem sites that regulate SNS activity after disease onset (25). Lastly, they support the need for a better understanding of how selective adrenergic treatments administered during acute and chronic phases directly affect joint pathology in IA.

**Figure 1:**
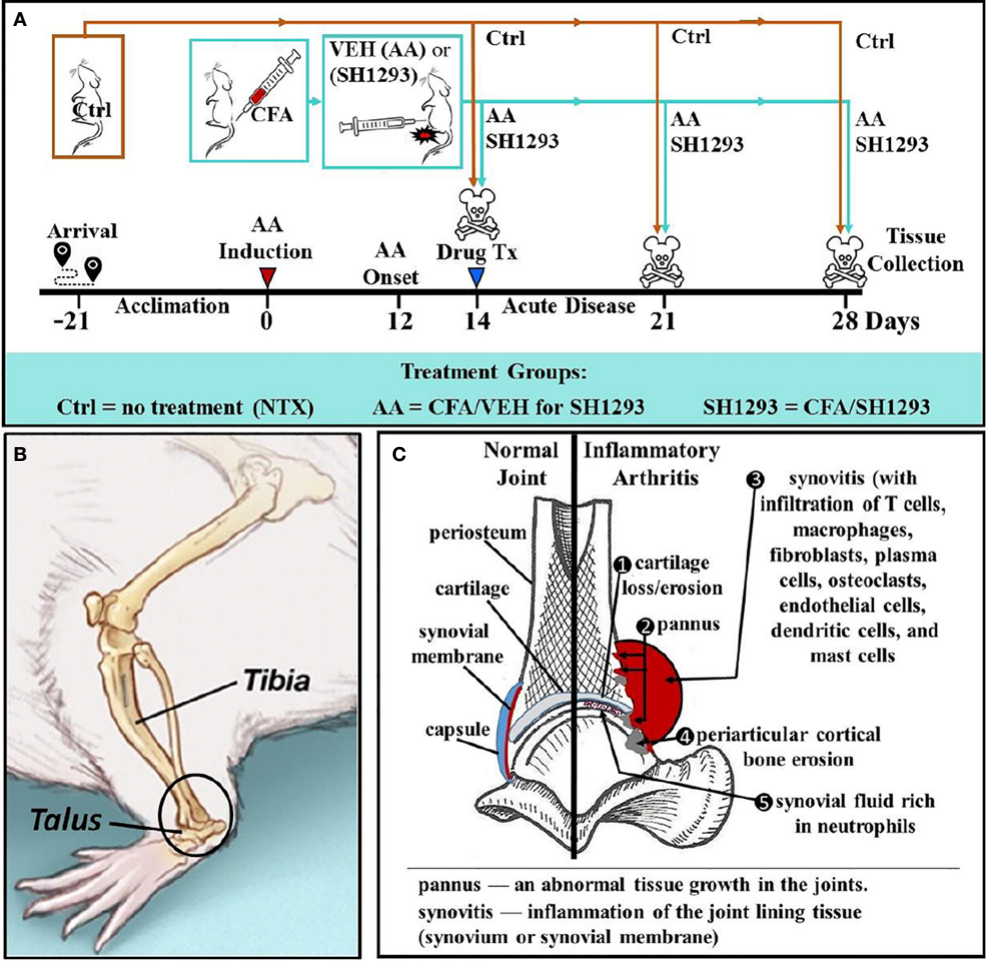
Experimental Design across Time **(A)**, Illustration of the Talocrural Joint **(B),** and Joint Pathology in IA **(C)**. **(A)** After acclimation to vivarium conditions for 21 day, arthritis was induced on D0 by injection of complete Freund’s adjuvant (CFA) into the base of the tail of young adult male Lewis rats to induce arthritis. Control rats (Ctrl) received no treatment. At D12, arthritic rats received the vehicle for SH1293 (AA) or SH1293. Rats were sacrificed D21 or D28 after disease induction and tissues were harvested. **(B)** A lateral view of the rat hind limb illustrates the talocrural joint formed by the distal tibia and talus. The region of interest (ROI) in this study is indicated in the open black circle. **(C)** As illustrated, arthritis-induced joint pathology was assessed in this study, including cartilage, bone erosion, and osteoclast number (1, 4), pannus formation (2), synovitis and cellular infiltration (3, 5). The density of sympathetic nerves was evaluated using the rate-limiting enzyme for norepinephrine synthesis, tyrosine hydroxylase, as a marker for these nerves.

Previous studies increased understanding of how α- and β_2_-ARs regulate immune response profiles in secondary immune organs to influence disease progression rather than the direct effects of these drugs in the hind limb. In these studies, disease outcome assessments of arthritis centered on footpad width and X-ray analysis of the hind limbs in arthritic animals (19). Similarly, clinical treatments currently target immune cells involved in disease progression, which often leaves patients immunocompromised. Targeting the SNS that regulates immune function could be a safer alternative treatment for RA that does not significantly compromise an immune response to pathogens. However, we don’t understand how sympathetic nerves influence joint pathology in AA or RA. Given the complex direct and indirect effects of the SNS in joint homeostasis, this study investigates the direct effects of SH1293 treatment on joint pathology after disease onset. In this study, AA in male Lewis rats are used as a model because it (1) induces severe arthritis, (2) does not require repeated immunization with CFA to induce it, (3) has well characterized phases of disease progression, and (4) is used previously to document disease sparing and immune-mediated effects of SH1293. SH1293 exerts its effects *via* α-adrenergic blockade and β_2_-adrenergic agonist activity. Drug-induced effects on AA are investigated by evaluating the histological changes in the distal tibia and the talocrural joint, a weight-bearing synovial joint connecting the distal end of the tibia with the proximal end of the talus (see **Figure 1**).

## Materials and Methods

### Supplies, Drugs and Adjuvant Preparation

The non-specific α-adrenergic antagonist, phentolamine and the selective β_2_-adrenergic agonist, terbutaline, were dissolved in 0.01 mM ascorbic acid in 0.9% sterile, endotoxin-free saline (all from Sigma Aldridge, St. Louis, MO, USA). CFA (0.03 g dried and heat-killed *Mycobacterium butyricum* (Difco, Detroit, MI, USA) was emulsified in 10 ml sterile mineral oil, and sonicated with a dismembraner (5 min), as previously described (19). A single preparation of CFA minimized variability; 100% of the animals developed arthritis.

### Animals and Induction of AA

**Figure 1A** illustrates the experimental design. Male Lewis rats (200–250 g; Charles River Laboratories, Raleigh, NC, USA) were housed two per cage for three weeks before starting each experiment. Animals were maintained on a 12-h off/on lighting schedule. Food (Purina Lab Diet 5001) and water were available *ad libitum*. Thirty-two male Lewis rats received 100 μl of CFA intradermally into the base of the tail to induce bilateral arthritis. For arthritic rats, food was placed in the cage floor, and water supplied using long-stemmed sipper tubes for easy access. All rats were observed to eat and drink throughout the study period. Animals were weighed biweekly. Other than arthritis development, rats were healthy throughout the experiment. The Animal Use and Care Committee approved all protocols before beginning the study, and the study complied with NIH guidelines.

### Experimental Design and Treatment Groups

Twelve days after arthritis induction (**Figure 1A**), rats were randomly assigned to one of three groups (*n* = 12): (1) untreated non-arthritic controls (Ctrl), (2) arthritic rats that received SH1293 (0.5 mg/day phentolamine and 1200 μg/day terbutaline, both drugs from Sigma) or (3) arthritic rats treated with the drug vehicle (AA). Drug treatments were divided into two injections administered intraperitoneally in a total volume of 250 µl per injection at 7 a.m. and 6 p.m. This drug regimen was based on previous reports demonstrating predictable pharmacological effects on disease severity in arthritic rats (19). Adrenergic therapies began at disease onset, D12 post-immunization, and continued through D21 or D28 (**Figure 1A**). On D11, beginning just prior to disease onset (D12), measurements of dorsoplantar footpad width in all treatment groups were measured and continued through sacrifice. X-ray and micro-computed tomography (micro-CT) were used to image the hind limbs (imaging view, and area of interest is shown in the circle in **Figure 1B**). Histological/histochemical techniques were employed to evaluate treatment-induced changes in arthritis-induced pathology in the distal tibia and talocrural joint (frontal view shown in **Figure 1C**). **Figure 1C** illustrates pathological changes in IA (right side) that were evaluated, and the normal components of the joint are shown on the left. Pathological changes evaluated were: (1) cartilage erosion, (2) pannus formation, (3/5) synovitis (inflammation with cellular infiltration), (4) trabecular and periarticular cortical bone erosion, (5) cellular infiltration into the synovial fluid and (6) sympathetic innervation (not shown).

### Assessment of Disease Severity

#### Dorsoplantar Footpad Widths

As previously described (18, 19), dorsoplantar widths of the hind feet were measured using a Mitutoyo Corporation dial thickness gauge every 2-3 days from D11 until sacrifice. Right and left footpad widths from each rat were averaged, and then mean footpad widths from rats within each group were averaged and expressed as a mean ± standard error of the mean (SEM). The hind limbs were photographed under anesthesia.

#### X-Ray Radiographic Analysis

Prior to sacrifice, radiographs of the hind limbs were taken using the following settings: 400 nN, 50 kVp, 0.4 s exposure time at 40 cm, using an X-OMAT processor. Using a grading scale as previously used (19), the radiographs were coded to obscure the treatment groups, then two independent observers subjectively rated each of the radiographs on the scale: 0 (normal), 1 (slight), 2 (mild), 3 (moderate), and 4 (severe) abnormalities in the tissue. Radiographs were scored for: (1) soft tissue edema as indicated by the width of soft tissue shadows and alterations in the normal configuration of the soft tissue planes; (2) osteoporosis (recognized by increases in radiolucency relative to uninvolved adjacent bone); (3) cartilage loss shown by narrowing of the joint spaces; (4) heterotopic ossification defined as proliferation of new bone tissue (fine ossified line paralleling normal bone, but not contiguous with calcified area of the bone itself); and (5) bone erosions. The radiographic scores for each category were added for both hind limbs giving a maximum score of 40.

### Animal Sacrifice and Tissue Preparation

On D28, rats were weighed before sacrifice by an overdose of 8% chloral hydrate (10 ml/kg body weight). After deep anesthesia, rats were perfused transcardially with phosphate-buffered saline (PBS) containing 0.5% sodium nitrite and then 250 ml of 4% paraformaldehyde in the same buffer (both pH 7.2). Right ankles were stripped of skin and muscle, immersed in fixative overnight at 4°C, washed with PBS, and decalcified in 10% ethylenediaminetetraacetic acid (pH 7.2) for 5 weeks at 4°C. Decalcified ankles were rinsed in deionized water (3 min), cut midsagittally from the calcaneus to the interspace between the second and the third phalanx and embedded in paraffin, cut side down. Tissue was sectioned at 5 μm for standard bone histology or 16 μm for immunohistochemistry for tyrosine hydroxylase (TH), the rate-limiting enzyme for norepinephrine synthesis. Tissue sections cut at 5 µm were mounted onto Fisher SuperFrost Plus slides (Statlab, Columbia, MD) for routine histology. Sections cut at 16 μm were mounted onto subbed slides (Vectabond reagent, Vector Laboratories Inc., Burlingame, CA) for immunohistochemistry.

Tissue sections were stained as follows. (1) Hematoxylin and eosin (H&E) staining was used to assess cellular lymphocytic infiltration; (2) tartrate-resistant acid phosphatase (TRAP) to visualize osteoclast lineage cells and assess bone area; (3) toluidine blue to characterize cartilage changes; and (4) trichrome staining to assess pannus formation, an abnormal layer of fibrovascular tissue or granulation tissue that grows in a tumor-like fashion over the joint surface. After staining, sections were dehydrated through a series of graded ethanol solutions, xylene-cleared, and cover-slipped with Cytoseal 28 (Stephens Scientific, Division of Richard Allan Scientific, Kalamazoo, MI). For immunohistochemistry, slides were cover-slipped with Acrymount (Statlab, Columbia, MD).

### Histological Staining and Evaluation

Histology was performed on the arthritic hind limbs to evaluated the distal tibia and the talocrural joint for hallmark features of IA (**Figure 1C**). Disease indices included: (1) cartilage loss/erosion; (2) pannus formation; (3) synovitis (infiltration of inflammatory cells into synovial membrane); (4) periarticular cortical bone erosion; and (5) infiltration of inflammatory cells into the synovial fluid. Ankle joints sectioned and stained with H&E or toluidine blue (Sigma) using standard laboratory procedures were used to evaluate lymphocytic infiltration or cartilage, respectively. TRAP staining was used to evaluate osteoclast numbers, and trichrome staining was used to assess the pannus. Because changes in density of sympathetic nerves alter ligand availability for ARs that consequently up- or down-regulate postsynaptic AR expression, sympathetic nerve density in the distal tibia was evaluated using immunohistochemistry for TH.

#### Staining for TRAP in Osteoclasts

TRAP-positive osteoclasts are responsible for bone resorption. Enzyme histochemistry for TRAP was performed to evaluate bone area and osteoclast numbers, as previously described (26) with some modifications. Dewaxed and hydrated sections were incubated for 1 h at 37°C in a TRAP staining solution freshly prepared as follows: pararosaniline chloride, dissolved in 2.3 N hydrochloric acid at 124 mM, was mixed to an equal volume (8 ml) of sodium nitrite (580 mM). To this solution, 12 ml of 0.1 M sodium acetate buffer (pH 5.0) was added with sodium tartrate (9.4 mM) and naphthol AS-triphosphate (691 µM; Sigma; N6125) dissolved in dimethylformamide. The solution was rapidly adjusted to pH 5.0 and used immediately. After washing with acetate buffer and distilled water, the sections were counterstained with methyl green and mounted with Permount (Fisher Scientific, Pittsburgh, PA).

#### Immunohistochemistry for TH

Tissue sections were stained for TH-positive (TH^+^) sympathetic nerves using a polyclonal rabbit anti-TH antibody (Chemicon International, Temecula, CA). Nickel sulfate intensification of the 3,3'-diaminobenzidine (0.04%; Sigma) was used to form a blue/black reaction product in TH^+^ nerves. All steps were carried out in 0.15 M PBS (pH 7.4) at 25°C using gentle agitation, unless otherwise indicated. Sections were rinsed in buffer and incubated for 30 min in 10% normal goat serum. The anti-TH primary antibody was diluted 1:500 in 0.15 M PBS containing 0.4% Triton X-100 and 0.25% bovine serum albumin (both from Sigma). Tissue was incubated in the primary antibody at 4°C for 24 h. Negative control sections were incubated under the same conditions without the primary antibody. On D2, sections were rinsed 6x10 min in PBS, incubated in 10% normal goat serum (30 min), and then in the secondary goat anti-rabbit antibody (Vector Elite kit) diluted in PBS (1:2,000; 90 min). Sections then were rinsed 4 x 10 min in buffer and incubated in 2.5% methanol with 8% hydrogen peroxide (30 min) to remove endogenous peroxidase activity. Following 6 x 10 min rinses, sections were incubated in an avidin-biotin-peroxidase complex (Vector Elite kit; 1:4,000 dilution in PBS; 90 min). Sections were rinsed 4 x 10 min in PBS buffer, followed by 2 x 10 min in 0.05 M acetate-imidazole buffer (pH 7.2), and developed in acetate-imidazole buffer containing 0.25 g/100 ml nickel (II) sulfate, 0.04 g/100 ml 3,3'-diaminobenzidine, and 0.005% hydrogen peroxide (15-20 min). All sections were rinsed 2 x 10 min in acetate-imidazole buffer, followed by 4 x 10 min rinses in PBS, mounted onto slides, and the slides cover-slipped, as described above.

Images were visualized and captured by digital photography with an Olympus BH-2 microscope. Calibration bars in all photographs represent 1 μm. RGB color images were captured and digitized at 200X using a high image resolution Olympus CCD video capture system at a resolution of 300 pixels per inch for automated measurement of the mean area of TH^+^ nerves in the joint capsule and synovial lining. In the joint capsule of three non-arthritic rats and five to seven arthritic rats, non-overlapping fields (200 x 100 µm or 20 mm^2^ per field) were captured to quantify sympathetic innervation using Image-Pro Plus^®^ imaging software (version 5.0; Media Cybernetics, Inc., Silver Spring, MD, USA). More fields were captured for analyses in arthritic rats, because the joint capsule volume is greater in arthritic than non-arthritic rats.

Sampling was blinded to the treatment group, and biased towards qualitatively selecting fields with the greatest density of nerve fibers. TH^+^ nerves were selected by the Image-Pro Plus software based upon their RGB color value and intensity. The staining intensities in all images were filtered by applying a threshold value to remove low-intensity pixels that represent nonspecific/background values. The threshold value was determined with mean and standard deviation pixel intensity values from a histogram analysis of all images sampled. This threshold process was applied in a consistent manner to all images to remove nonspecific background staining. Non-neural TH^+^ staining was excluded based on profile size and/or their non-linear profile. Data were expressed as a percent area of TH^+^ nerves per field. All fields per rat were averaged, and the mean percent area of TH^+^ nerves ± SEM for each treatment group was calculated as a mean of a mean.

In H&E-stained tissue, lymphocyte infiltration throughout the whole ankle section was examined. The site for sampling of the inflammatory cell infiltration in the distal tibia is demonstrated in **Figure 1B**, (open black box). Cellular infiltration was evaluated using a grading scale with +, ++, or +++, representing low (<300 lymphocytes), moderate (~300 to 600 lymphocytes), or high cellular infiltration (>900 lymphocytes), respectively.

Images from trichrome-stained sections were captured by digital photography with an Olympus BH-2 microscope and analyzed with Image-Pro Plus^®^ software. Trichrome-stained images were used to determine the percentage of pannus in the tissue section. The whole section was used because of the pervasive nature of the pannus. Trichrome-stained sections were used to normalize the amount of collagen outside of the bone capsule and ligaments to control values. For the toluidine blue-stained tissue sections, the area of the articular cartilage in the distal tibia and trochlea of the talus were traced in each tissue section, and quantified using the semiautomated Osteomeasure image analysis system and its digitizing pad to obtain the total area in the tissue sections (OsteoMetrics, Inc., Atlanta, GA). The total mean cartilage area was expressed in mm^2^.

TRAP-stained sections were observed for bone area and analyzed with the semiautomated OsteoMeasure image analysis system and digitizing pad. Three sample sites (zones 1 through 3), each consisting of a 350 x 350 μm^2^ area, were examined from the distal tibia of each rat. Zone 3 (closest to the joint) encompassed tibial articular cartilage/cartilage remnants and the subchondral bone located proximally. Zone 2 encompassed any subchondral bone and trabecular bone. Zone 1, most distal to the talocrural joint, encompassed trabecular bone located most superiorly in the sampling site. The bone present in each zone was outlined using the digitized pad and the area was then computed using Image-Pro Plus^®^. TRAP-positive osteoclasts were also counted in these zones.

### 3-D Image Analysis Using Micro-CT

The left ankles of rats were imaged using microcomputed tomography (MicroCAT II, Siemans Medical Solutions USA, Inc., PA) to determine tibial bone volume within a region of interest (ROI) using an Amira software program (Visage Imaging, Inc., Carlsbad, CA). Imaging equipment was an 80keV X-ray source. The micro-CT camera used a 2048^2^ matrix, 4x4 binning with an exposure time of 200 ms. The X-ray parameters were 75 kVp at 100 µA with a 0.5 mm Al filter. The CT scan included 360 rotation steps for a total scan time of 10 min and 45 sec. The reconstructed 3D image was 512 for a voxel size of 0.126 μm^3^.

The distal tibias were evaluated for thickness by quantifying the number of slices from medial to lateral. From this measurement, the middle of the tibia was determined. By using the Point Probe function in the Amira program, it was possible to discern a difference between bone and soft tissue by selecting a spherical ROI with a radius of 1 mm just proximal to the apex of the distal articular cartilage in the middle slice.

### Statistical Analyses

For all morphometric analyses, the examiners were blinded to the treatment groups. Where appropriate, histological data were expressed as a mean ± SEM and treatment groups were compared using one-way analysis of variance (ANOVA) with subsequent post-hoc comparison performed using the Tukey method for significant differences (*p <* 0.05) as determined by ANOVA. All *p* values were reported from post-hoc comparisons (*p <* 0.05, *; *p <* 0.01, **, *p <* 0.001, ***, *p <* 0.0001, ****).

## Results

### SH1293 Reduced Footpad Widths in Arthritic Rats

**Figure 2:**
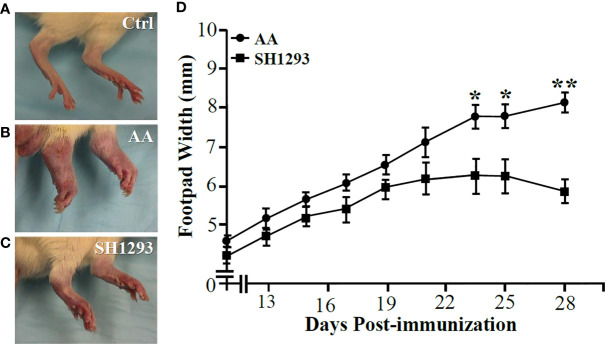
SH1293 Suppressed Arthritis in the Hind limbs **(A–C)** and Reduced Footpad Widths **(D)**. **(A–C)** Hind feet images at the study endpoint (D28) from non-treated controls [Ctrl; **(A)**], vehicle-treated arthritic rats [AA; **(B)**], and SH1293-treated arthritic rats [SH1293; **(C)**]. **(A)** Non-arthritic Ctrl rats illustrate normal appearance of the hind limbs. **(B)** Vehicle-treated AA rats developed severe arthritis with severe swelling, erythema, joint stiffness, and deformities of the ankle and foot joints. **(C)** Arthritic rats treated with SH1293 developed a less severe arthritis, based on reduced swelling, erythema, reduced joint stiffness, and less hind foot deformities compared with vehicle-treated AA rats. **(D)** This graph plots the dorsoplantar foot pad widths (expressed as a mean of the right and left foot pad) from male Lewis rats across time after immunization with CFA to induce arthritis. Disease onset occurred at D12; severe disease is established at D28. Mean dorsoplantar widths increased through D21; no significant differences in mean dorsoplantar footpad widths was observed between AA and SH1293-treated rats before D22. By D24, soft tissue swelling was significantly less in SH1293-treated compared with vehicle-treated AA rats, a finding that continued through D28. Non-arthritic Ctrl rats maintained an average footpad with of 20 ± 0.2 mm (data not shown). Graphed data are presented as mean dorsoplantar width in millimeters ± SEM with an *n* of 8 rats per treatment group. Kruskal-Wallis analysis of the data was followed by Dunn post-hoc testing (*, *p* < 0.05; **, *p* < 0.01).

The hind limbs from Ctrl animals are shown in **Figure 2A** for comparison. All animals treated with CFA developed arthritis with disease onset between 10 to 12 days after the treatment (**Figures 2B, C**). In AA rats, dorsoplantar paw thickness increased (**Figure 2B**). Treatment of arthritic rats with SH1293 (SH1293) reduced disease severity (**Figure 2C**). Mean footpad widths from AA and SH1293 groups across time are plotted in **Figure 2D**. In vehicle-treated arthritic AA rats, dorsoplantar widths progressively rose through the acute and chronic phases of the disease compared with Ctrl rat (**Figure 2D**; Ctrl not shown). SH1293 had no acute effect that delayed disease onset (D12), but treatment with SH1293 initiated at disease onset significantly reduced soft tissue swelling of the foot pads post-immunization with CFA across time compared with AA rats (**Figure 2D**). Thus, SH1293 flattened the curve across time. Significant differences in dorsoplantar footpad widths were realized by D23 post-immunization between AA and SH1293 rats, and continued to the end of the study (D23 or D25: *, *p <* 0.05; D28: **, *p <* 0.01). These findings were replicated twice with similar findings. Representative hind paws of vehicle (AA) and SH1293-treated arthritic rats (SH1293) and non-treated rats (Ctrl) on D28 shown in **Figures 2A–C** illustrate the differences in disease severity at the study endpoint.

### SH1293 Increased Subcapsular Osteophyte Formation and Reduced Bone Marrow Inflammation

**Figure 3:**
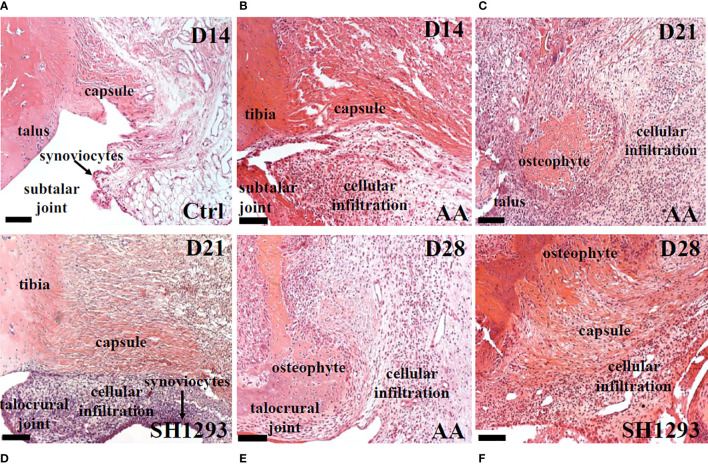
SH1293 Reduced Inflammatory Infiltrates in Talus and Subtalar Joint. Representative images of H&E-stained sections of the ankle joint from Ctrl, AA and SH1293 rats. **(A)** In Ctrl rats, fibrous connective tissue forming the capsule of the subtalar joint was intact and periarticular adipose tissue was a prominent feature. Synoviocytes formed a thin synovial lining between the joint capsule and the joint cavity and was largely devoid of a cellular infiltrate. **(B)** By D14, joints from AA rats revealed that collagen fibers of the capsule remained intact in the subtalar joint, however, a thickening of the synovial capsule and cellular infiltration by leukocytes were apparent. **(C)** Arthritis progressed in joints from AA rats at D21 as indicated by continued thickening of the synovial membrane, a severe increase in cellular infiltration, distal tibial bone resorption and the growth of large areas of osteophyte. **(D)** In contrast to vehicle treated AA rats at D21, arthritis proceeded more slowly in rats treated with SH1293. In this treatment group, collagen fibers forming the capsule remained intact, but proliferation of synoviocytes was apparent with a thickening of the synovial membrane and leukocyte infiltration. While synovial thickening and leukocyte infiltration was present, it was much less than that observed for untreated AA rats. **(E)** By D28 in vehicle-treated AA rats, disease continued to progress as evident by the extensive distal tibial bone resorption, the presence of large osteophyte formation and increased leukocyte infiltration. **(F)** By D28 in SH1293-treated AA rats, disease continued to progress but at a slower rate than vehicle treated rats. Some distal tibial bone resorption and osteophyte formation was now apparent and a greater leukocyte infiltration was observed than at D21 for this treatment group. Calibration bars = 100 µm.

In H&E-stained sections of the talocrural joint, general histology and inflammation were evaluated **(** **Figures 3A–F** **)**. In Ctrl rats, the capsule of the talocrural joint was continuous with the periosteum, and enveloped the synovial joint space, with an outer and inner layer of avascular fibrous membranes of dense irregular and loose fibrous connective tissue, respectively (**Figure 3A**). The inner synovial membrane, lined with synoviocytes, encased the joint space. Lymphocytic infiltration was low, but evident in the connective tissue beneath the synovial membrane and the adipose tissue (**Figure 3A**). In AA rats, on D14, collagen fibers remained intact in the capsule of the talocrural joint, but lymphocyte infiltration markedly increased in the talocrural joint, and appeared to replace adipose tissue (**Figure 3B**). By D21, prominent features of the distal tibia of AA rats were marked bone resorption, formation of large bone spurs or osteophytes, severely increased lymphocytic infiltrates, depletion of fat cells, and loss of a clearly defined capsule (**Figure 3C**). In SH1293 rats, osteophytes and bone erosion were not present (**Figure 3D**). Instead collagen fibers forming the capsule remained intact and a clearly defined talocrural joint was evident at D21. An increase in cellular infiltrates was present within the synovium (**Figure 3D**). At D28, the talocrural joints of AA rats displayed more marked bone resorption, accompanied by areas of large osteophytes, indicating new bone formation, and less influx of lymphocytes in the breached capsule than at D21 (**Figure 3E**). In SH1293 rats, there was greater bone preservation and more extensive osteophyte formation in the distal tibial bone compared with AA rats (**Figure 3F**). SH1293 also reduced inflammatory cell infiltration at D28 compared with AA rats (**Figures 3E, 3F**, respectively).

**Figure 4:**
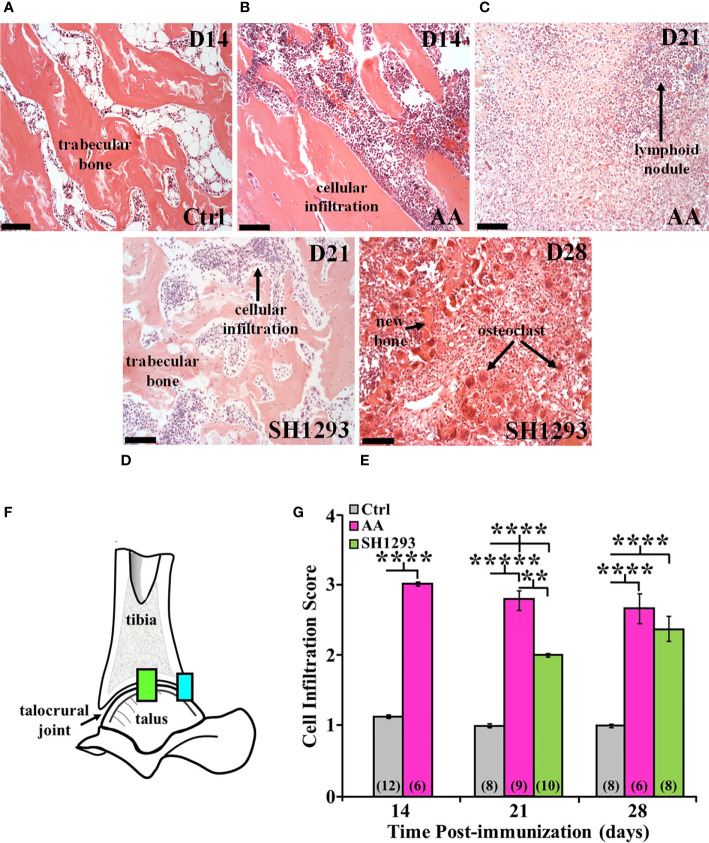
SH1293 Suppresses Cellular Infiltration into the Distal Tibia and Proximal Talus of Arthritic Rats. H&E images from the distal tibia and proximal talus of Ctrl, AA, and SH1293 rats revealed striking differences in inflammatory cell infiltration among the trabeculae in the bone marrow spaces between experimental groups. Calibration bars = 100 µm. **(A)** In Ctrl rats, trabecular bone in the distal tibia just proximal to the subcortical bone was intact and appeared normal with a sparse presence of leukocytes. **(B)** On D14, in the distal tibia of vehicle treated AA rats, trabecular bone was intact, however, leukocyte migration into the bone tissue was apparent as shown here. **(C)** By D21, extensive trabecular bone resorption was accompanied by extensive cellular infiltration in AA rats. **(D)** On D21, trabecular bone was preserved to a greater extent in SH1293 rats compared with AA rats at this time point. Cellular infiltration was apparent, but its magnitude was comparable to that in AA rats on D14. **(E)** By D28, trabecular bone resorption is more evident in SH1293 rats and consistent with a greater presence of osteoclasts. Inflammatory leukocytes are present in greater numbers compared with rats treated with SH1293 at D21. **(F)** Morphometric analysis was completed at two evaluation sites within the talocrural joint, as illustrated in this diagram. **(G)** Cellular infiltration was scored and graphed across time after immunization in days. The numbers in parentheses in each bar indicate the number of rats per group. Asterisks mark statistically significant differences (**p < 0.01, ****p < 0.0001).

Trabecular bone was intact just proximal to subcortical bone in the distal tibia of Ctrl rats (**Figure 4A**). In AA rats, inflammatory cells infiltrated into trabecular bone among the trabeculae in the distal tibia at D14 (**Figure 4B**) compared with their sparse presence in this compartment in Ctrl rats (**Figure 4A**). The volume of trabecular bone in AA rats (**Figure 4B**) was reduced compared with non-arthritic Ctrl rats (**Figure 4A**), based on an increased spacing between trabecular bone. By D21, there was complete trabecular bone resorption and formation of lymphocytic-like nodules in AA rats (**Figure 4C**, long arrow). Trabecular bone was better preserved in SH1293 rats, and cellular infiltration was reduced at D21 (**Figure 4D**). Notable at D28, inflammatory cell infiltration in SH1293-treated rats was less marked than in AA rats at D21 (**Figure 3C**), and there was evidence of active bone remodeling based on new bone formation and the presence of osteoclasts (**Figure 4E**, short arrow and long arrows, respectively). At D28, bone volume in SH1293 rats was reduced compared with Ctrl rats, despite new bone formation (**Figure 4E**, short arrow).

Morphometric analysis at two evaluation sites within the talocrural joint (**Figure 4F**) at D14 confirmed a 3-fold increase (****, *p* < 0.0001) in lymphocytic infiltration in AA compared with Ctrl rats (**Figure 4G** **)**. At D21 post-challenge, the lymphocytic infiltration score remained elevated (*****, *p <* 0.00001) in AA rats compared with Ctrl rats (**Figure 4G**). Lymphocytic infiltration in SH1293 rats was also significantly greater (****, *p <* 0.0001) than in Ctrl rats (**Figure 4G**), but less than in AA rat (**, *p* < 0.01). SH1293 reduced mean lymphocyte scores by 28.6% compared with AA rats on D21 (**Figure 4G**). By D28, both AA and SH1293 rats had significantly greater (****, *p <* 0.0001) mean lymphocytic infiltration scores than scores from Ctrl rats (**Figure 4G**). SH1293 treatment reduced the mean lymphocyte score by 11.2% at D21 compared with scores in AA rats. At D21, lymphocytic infiltration negatively correlated (*r =* -0.78) with the density of sympathetic nerves in Ctrl rats. At D28, the extensiveness of lymphocytic infiltration inversely correlated (*p <* 0.05) with sympathetic nerve density (*r =* -0.71) in SH1293 rats.

### Micro-CT and X-Ray Analyses Support Anti-Arthritic Effects of SH1293 on Bone Resorption

**Figure 5:**
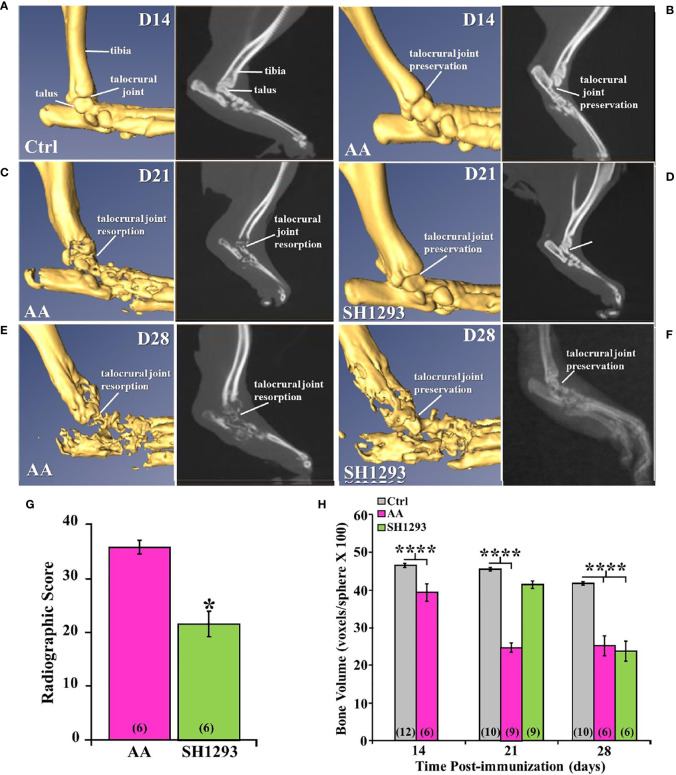
Representative Micro-CT (left panels) and corresponding X-ray (right panels) images of the talocrural joint in the ankle of control (Ctrl) and arthritic rats (AA) with or without adrenergic drug treatment across time in days **(D)**. **(A)** Ctrl rats display intact joint structure and normal bone surfaces based on micro-CT (left) and radiographic (right) images of the hind limbs. **(B)** At D14, the talocrural joint structure was similar in AA and Ctrl rats based on micro-CT (left) and X-rays (right). The trochlea of the talus and articular surface of the tibia had regular borders and smooth articular surfaces. There were no signs of bone erosion or osteophyte formation at this time point. **(C)** By D21, arthritic changes in bone structure typical of RA are evident in the CT (left) and X-ray (right) images. The ankle joint and metatarsophalangeal joint exhibit bone erosions, bone resorption, narrowing of the joint spaces, soft tissue swelling and remodeling of the distal tibia, talus, and proximal foot in AA rats (left panel). X-ray (right) also reveal joint destruction, joint displacement and irregular bony proliferation in the distal tibia, talus and other small bones of the foot. **(D)** In contrast, SH1293 treated AA rats had remarkable sparing of joint destruction on D21 based on micro-CT (left) and X-ray (right) images. Soft tissue swelling was also reduced comparted to vehicle-treated AA rats. In fact, images more closely resembled those from Ctrl rats. **(E)** In AA rats on D28, CT (left) and X-ray (right) scans showed severe bone resorption in bones that form the talocrural joint and metacarpals of the foot. Periosteal bone formation resulting in joint space narrowing was evident at D28. **(F)** In SH1293 rats at D28, bone resorption near the talocrural joint, periosteal bone formation and joint space narrowing was still strikingly less than in AA rats treated with vehicle, but did progress substantially compared with Ctrl rats. **(G)** Radiographic scoring revealed a significant decrease in joint destruction in SH1293 rats compared with AA rats (*, *p <* 0.05). **(H)** Bone volume was significantly reduced in AA rats compared with Ctrl rats on D14 (****, *p <* 0.0001), D21 (****, *p <* 0.0001), and D28 (****, *p <* 0.0001). SH1293 had a sparing effect on bone volume at D21, as it did not differ from Ctrl rats. However, by D28 the sparing effect was lost (****, *p <* 0.0001). The *n*’s for each group are indicated in parentheses at the base of each bar of the graphs **(G, H)**.

Micro-CT and radiographic images of the hind limbs from Ctrl rats had intact joint structure and normal bone surfaces (**Figure 5A**, left and right panel, respectively). At D14, micro-CT scans and radiographs (left and right panels, respectively) from AA (**Figure 5B**) and Ctrl (**Figure 5A** **)** rats were similar. The integrity of the talocrural joint appeared preserved in AA rats (**Figure 5B**); its structure was similar to that seen in Ctrl rats. The trochlea of the talus and the articular surface of the tibia had regular borders and smooth articular surfaces, and bone erosion and osteophyte formation were absent. By D21 post-CFA challenge, CT and X-ray images revealed typical arthritic changes evident in AA rats (**Figure 5C**, left and right panels, respectively). Disease progressed compared with that observed at D14, with ankle joint and metatarsophalangeal joint erosion, bone resorption, narrowing of the joint space due to soft tissue swelling, and remodeling of the distal tibia, talus, and proximal foot in AA rats (**Figure 5C**). Joint destruction, joint displacement, and irregular bony proliferation in the distal tibia, talus, and other bones of the ankle were evident. At D21, SH1293 rats (**Figure 5D**) had a marked sparing effect on bone loss and soft tissue swelling to the extent that the images most resembled the images of the hind limbs of Ctrl (**Figure 5A**) and AA rats at D14 (**Figure 5B**). Treatment with SH1293 protected against bone destruction/resorption, and preserved the architecture of the affected hind limb; there was complete preservation of the talocrural joint.

**Figure 5E** demonstrates representative findings in AA rats at D28 (chronic disease phase). There was severe bone resorption in the ankle and tarsal bone resorption in AA rats (**Figure 5E**), but the findings across animals were more variable than at the previous time points. Bone degradation and soft tissue swelling became strikingly more severe in AA rats (**Figure 5E**) than in AA rats at D21 (**Figure 5C**); there was severe destruction of the distal tibia, the talus and proximal bones of the foot at this time point (acute disease). At D28, SH1293 rats (**Figure 5F**) had greater bone density near the talocrural joint and in the tarsal bone than in AA rats (**Figure 5E**). Articulation of the bones that form the talocrural joint were not evident in AA rats (**Figure 5E**), but were present in SH1293 rats (**Figure 5F**). Periosteal bone formation led to a narrowing of the spaces between the tarsal and the talus bones and between the metatarsals. Preventative effects of SH1293 on soft tissue swelling were reduced at D28 compared with that seen at D21, but SH1293 slowed disease progression, based on CT scans and X-rays from AA rats at D28. Periosteal bone formation narrowed the space between the tarsal and the talus and between the metatarsals, and reduced bone radiolucency progressed in rats treated with SH1293 between D21 and D28, but to a lesser extent than in AA rats. Scoring of radiographs (**Figure 5G**) quantified the joint destruction in AA rats at D28, revealing a reduction (*, *p* < 0.05) in joint destruction in SH1293 rats compared with AA rats. Loss of subchondral bone volume (**Figure 5H**) was evident in AA rats by D14 (****, *p*<0.0001), and progressed further by D21 (****, *p* < 0.0001) compared with Ctrl rats. SH1293 delayed AA-induced bone loss at D21, as bone volume did not differ from Ctrl rats. At D28, bone volume in both AA and SH1293 rats was reduced to comparable levels (**Figure 5H**), and was significantly lower than in Ctrl rats (****, *p* < 0.0001).

### SH1293 Reduced Osteoclast Number and Bone Area in the Distal Tibia and Talocrural Joints of Arthritic Rats

**Figure 6:**
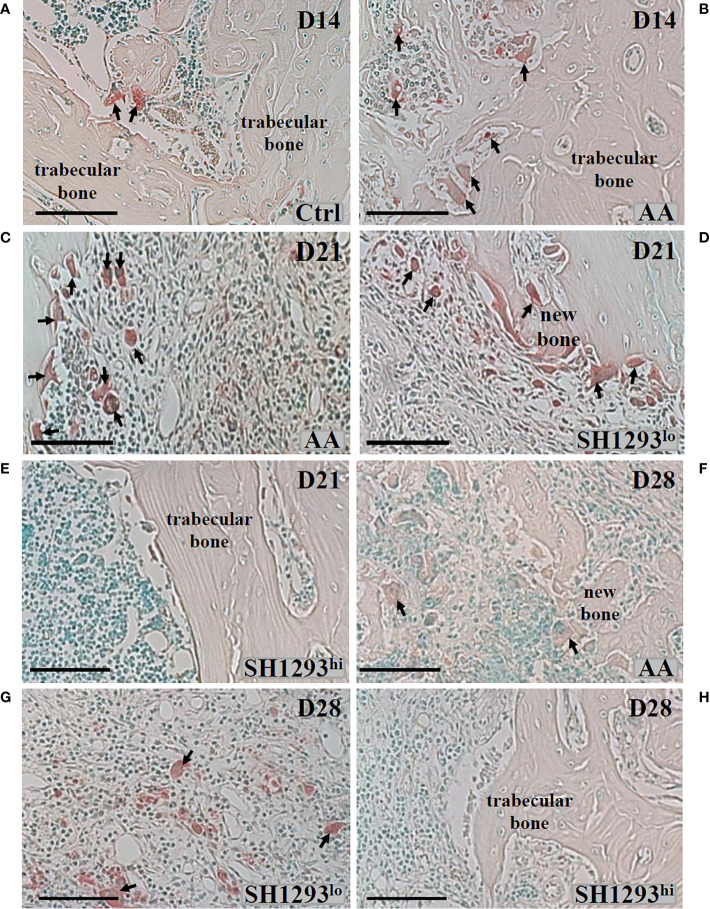
SH1293 Minimizes Osteoclast Destruction of Trabecular Bone in High Responding-arthritic Rats by Reducing the Number of Infiltrating Osteoclasts. Representative photomicrographs of TRAP-stained tissue sections from zone 2 (see **Figure 7A**). **(A)** In Ctrl rats, osteoclast activity was minimal, a finding consistent with the normal appearance and abundance of trabecular bone in Ctrl rats on D14. Arrows indicate only two sites of osteoclast activity. **(B)** On D14, osteoclast activity was more apparent in AA rats, with more osteoclasts (arrows) present than observed in Ctrl rats, but trabecular bone appeared similar to that observed in Ctrl rats. **(C)** By D21, osteoclasts were abundant, and their increased presence consistent with severe trabecular bone resorption in AA rats **(D, E)**. In SH1293 rats, we observed SH1293^lo^- and SH1293^hi^-responding rats on D21, **(D, E)**, respectively. In low-responding SH1293^lo^ rats **(D)**, osteoclast presence and the resorption of trabecular bone was similar to that observed for vehicle-treated AA rats at this time point. In high-responding SH1293^hi^ rats **(E)**, the absence of osteoclasts and preservation of the trabecular bone was remarkable. **(F)** In AA rats on D28, osteoclasts were more numerous than at D21. Trabecular bone resorption was greater than that observed at previous time points and the deposition of new bone was observed in this treatment group. **(G)** On D28, in SH1293^lo^ rats, osteoclast numbers and trabecular bone loss were similar to that observed for AA rats. **(H)** In contrast, in SH1293^hi^ rats on D28, osteoclasts were not observed in trabecular bone and had remarkable sparing of trabecular bone. Calibration bar = 100 μm.

Bone mass is controlled by a balance between osteoclast and osteoblast activity. Mature osteoclasts release acidic enzymes into the microenvironment of the bone, including TRAP (27). The uncontrolled release of TRAP and related enzymes drives bone erosion and deformation (28, 29). For these reasons, TRAP was used as a marker for osteoclast infiltration and bone erosion/remodeling. The talocrural joint from Ctrl rats is shown in **Figure 6A**. As expected, neither the subchondral bone nor the articular cartilage showed any histological evidence of resorption; osteoclast activity was miniscule. In AA rats, subchondral bone resorption is apparent at the apex of the distal tibial concavity at D14 (**Figure 6B**), but articular cartilage at this site was preserved (**Figure 6B**). A few osteoclasts were present, and trabecular bone was largely preserved. At D21, osteoclasts were abundant in AA rats, and trabecular bone was severely resorbed (**Figure 6C**). Interestingly, a bimodal response was observed in rats treated with SH1293. SH1293 failed to preserve trabecular bone in five rats (designated SH1293^lo^). In these rats, bone loss was accompanied by an increase in osteoclasts, and abnormal laying down of new bone (**Figure 6D** **)**. In contrast, seven rats showed bone-sparing effects of SH1293 (designated SH1293^hi^) with remarkable preservation of trabecular bone (**Figure 6E**). No resorption of either subchondral bone or articular cartilage was evident in SH1293^hi^ arthritic rats, and osteoclasts were sparse (**Figure 6E**) similar to the Ctrl group. At D28, osteoclasts were present among remnants of trabecular bone and the deposition of new bone in AA rats (arrows, **Figure 6F**). Differences in treatment responses to spare osteoclast driven bone destruction between SH1293^lo^ and SH1293^hi^ rats remained discernible at D28 (**Figures 6G, H**, respectively). Bone structure was similar in SH1293^lo^ rats to that seen in AA rats at this time point. In SH1293^hi^ rats, trabecular bone remained intact and osteoclasts were sparse.

**Figure 7:**
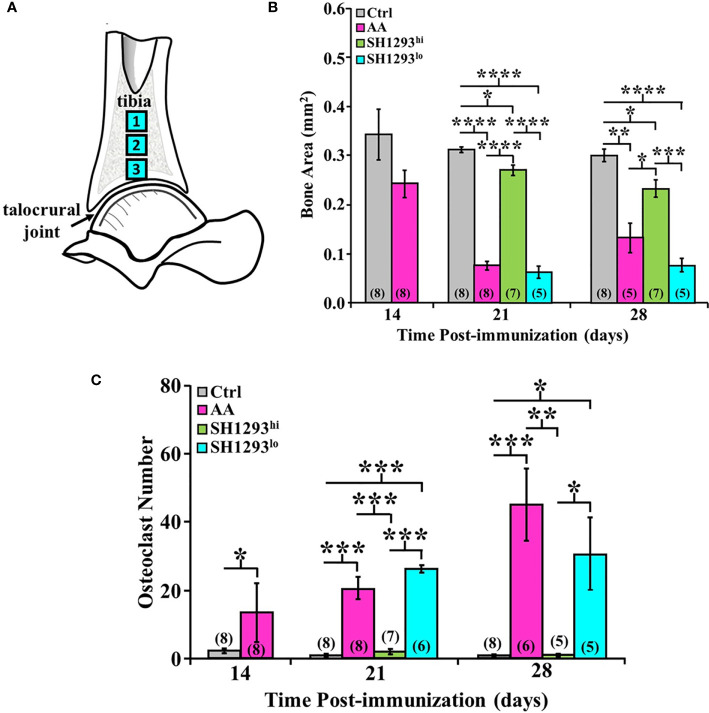
High and Low Response of SH1293 on Bone Area and Osteoclast Numbers in the Distal Talus. **(A)** Schematic illustration of the sampling sites in distal tibia used to quantify mean bone area (in mm^2^), and mean osteoclast number. **(B)** Bone area is plotted as a mean of a mean in mm^2^ across time (in days) after immunization to induce IA. Bone area in AA rats did not differ from Ctrl rats at D14. Although significance levels between treatment groups differed at D21 and D28, the findings at these time points were similar. At D21 and D28, bone area was significantly less in AA than Ctrl rats. In contrast, at D21 and D28, bone area was significantly greater in SH1293^hi^-responder than in AA or SH1293^lo^-responder rats. Still, bone area in SH1293hi rats was significantly less than in Ctrl rats. **(C)** Graph plotting the number of osteoclasts expressed as a mean of a mean in three sample sites in the distal tibia across time (in days) after immunization to induce IA. Despite the variability in AA rats, mean osteoclast number was greater in AA than Ctrl rats at D14. At D21 and D28, mean osteoclast number was low in Ctrl and SH1293^hi^-responders compared with their number in the distal tibia of AA or SH1293^lo^-responder rats. The numbers in parentheses at the bottom of each bar indicate the number of rats per group. Asterisks identify statistically significant differences as follows: *p < 0.05, **p < 0.01, ***p < 0.001, ****p < 0.0001.

Bone area and osteoclast number was morphometrically assessed at D7, D14, and D28 in three sites of the distal tibia. As shown in the schematic in **Figure 7A**. High- and low-responders of SH1293 were identified for its effects on bone area and osteoclast number at D21 and D28 (**Figures 7B, C**, respectively). At D14, mean bone area did not significantly differ in AA and Ctrl rats (*p >* 0.05). By D21, bone area was significantly reduced in AA, SH1293^hi^, and SH1293^lo^ compared with Ctrl rats (****, *p <* 0.0001; *, *p* < 0.05; and ****, *p* < 0.0001, respectively). However, mean bone area in SH1293^hi^ rats was significantly higher (****, *p <* 0.0001) than in AA and SH1293^lo^ rats. On D21, mean bone area in high responders was 74.1% greater in SH1293^hi^ rats than in AA rats, but was reduced 14.3% in SH1293^lo^ rats compared with AA rats (**Figure 7B**). A similar pattern was seen at D28. AA, SH1293^hi^, and SH1293^lo^ rats had significantly lower mean bone area than the Ctrl rats (**, *p <* 0.01; *, *p* < 0.05; and ****, *p <* 0.0001, respectively). SH1293^hi^ rats continued to have higher bone area than in AA and SH1293^lo^ rats (*, *p <* 0.05 and ***, *p <* 0.001, respectively; **Figure 7B**). The SH1293 group was not divided into high or low responders for correlational analysis. At D21, a direct correlation between bone area and sympathetic nerves (*r =* 0.84) was observed in rats treated with SH1293. At D28, the mean bone area in SH1293 rats inversely correlated with lymphocytes (*r =* -0.83), and bone area correlated with sympathetic nerves (*r =* 0.93).

Regarding osteoclasts, at D14, there was a significant increase (***, *p <* 0.001) in mean osteoclast number in AA compared with Ctrl rats (**Figure 7C**). Osteoclasts were sparse in Ctrl rats, averaging 2.27 ± 1.91 cells per three zones, whereas AA rats averaged 13.4 ± 19.40 cells per three zones. Mean osteoclast numbers were similar in AA rats at D14 and D21, although within-group variability was greater at D14 (**Figure 7C**). At D21, more osteoclasts were present (***, *p* < 0.001) in AA or in SH1293^lo^ than in SH1293^hi^ rats. A similar pattern was observed for osteoclast number in each treatment group at D28 and D21, although osteoclast numbers were higher in AA rats at D28. At D28, osteoclasts were more abundant in AA than in Ctrl or SH1293^hi^ rats (***, *p* < 0.001, or **, *p* < 0.01, respectively) (**Figure 7C**). Osteoclast number was also greater (*, *p* < 0.05) in SH1293^lo^ than Ctrl or SH1293^hi^ rats. At D28, there was no significant difference (*, *p* > 0.05) in osteoclast number between the Ctrl and SH1293^hi^ groups (**Figure 7C**). By D28, the AA rats averaged 45.2 cells per 3 zones (± 10.8), the SH1293^hi^ rats averaged zero cells per 3 zones (± 0.0), and the SH1293^lo^ rats averaged 30.8 (± 10.9) cells per 3 zones, while the Ctrl rats averaged 0.5 cells per 3 zones (± 0.5). These findings are consistent with bone preservation observed for SH1293^hi^ rats compared with bone area loss observed in the AA and SH1293^lo^ rats (**Figure 7B**).

### SH1293 Preserved Articular Cartilage in Arthritic Hind Limbs, Had No Effect on Pannus Formation, But Depleted Subchondral Bone and Intraarticular White Fat

**Figure 8:**
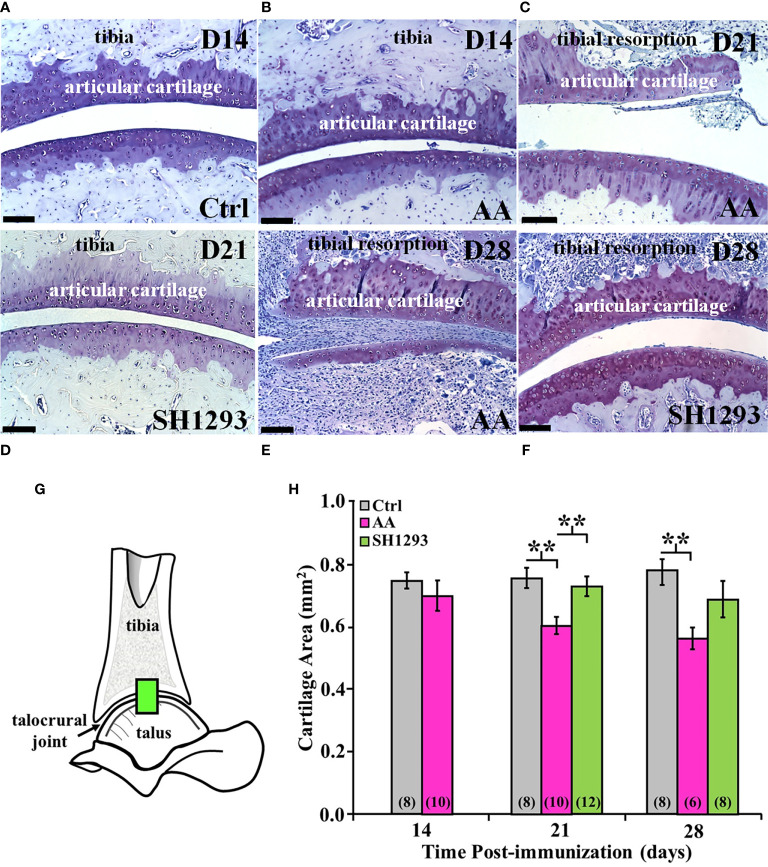
SH1293 Spared Articular Cartilage Resorption in AA Rats. Resorption of articular cartilage and subchondral bone were assessed at the apex of the distal tibial concavity. **(A–F)** Toluidine blue-stained images from the distal tibia and proximal talus of Ctrl, AA, and SH1293 rats revealed striking differences in articular cartilage and subchondral bone between experimental groups across time. Calibration bars = 100 µm. **(A)** In Ctrl rats, articular cartilage and subchondral bone were normal. **(B)** In AA rats at D14, AA induced resorption of the subchondral bone, but the articular cartilage appeared normal. **(C)** In AA rats at D21, subchondral bone was resorbed and articular cartilage lost. **(D)** At D21, there was no discernable changes in the articular cartilage or subchondral bone in SH1293-treated rats. **(E)** In AA rats, resorption of both the subchondral bone and articular cartilage was severe in the distal tibial concavity by D28. **(F)** The sparing effects of SH1293 observed on D21 in SH1293-treated rats on cartilage and subchondral bone resorption were lost by D28. **(G, H)** Morphometric analysis of cartilage area **(H)** was completed in a zone within the talocrural joint, as illustrated in the diagram of the talocrural joint **(G)**. **(H)** Cartilage area did not differ between Ctrl and AA rats at D14. However, by D21, AA rats had significantly reduced cartilage area compared with both the Ctrl (**p < 0.01) and SH1293 (**p < 0.01) groups. At D28, cartilage area in AA rats was less than that observed for Ctrl rats (**p < 0.01), but cartilage area did not differ between SH1293 and Ctrl rats. The *n*’s per group are indicated in parentheses at the base of each bar.

Subchondral bone, cartilage at the apex of the distal tibial concavity, and adipose tissue was preserved in Ctrl rats at D14 (**Figure 8A**). However, in AA rats, subchondral bone was resorbed and extra-articular white adipose tissue (analogous to Kager’s fat pad in humans) was not present at D14. Articular cartilage at the apex of the distal tibial concavity was preserved in AA rats at this time point (**Figure 8B**). At D21, resorption of subchondral bone progressed, and articular cartilage resorption at the apex of the distal tibial concavity was evident in AA rats (**Figure 8C**). In SH1293 rats, there was no resorption of the subchondral bone or articular cartilage at the distal tibial concavity on D14 (data not shown) and on D21 (**Figure 8D**). By D28, subchondral bone and articular cartilage was lost in AA rats at the distal tibial concavity (**Figure 8E**), but in SH1293-responder rats (**Figure 8F** **)** subchondral bone resorption and articular cartilage resorption of the distal tibial concavity was minimal.

At D14, there was no significant difference in the mean articular cartilages in the sampled site (**Figure 8G**) in the talocrural joint between the Ctrl and AA rats (**Figure 8H**). By D21, mean articular cartilage area was reduced (*p <* 0.01) in AA rats compared with Ctrl rats. Treatment with SH1293 begun after disease onset prevented the CFA-induced cartilage loss. Mean articular cartilage area was significantly greater (*p <* 0.01) in SH1293 rats than in AA rats. Mean articular cartilage in the talocrural joint was comparable in SH1293-treated and Ctrl rats. At D28, no further loss of articular cartilage occurred in AA rats, based on mean values that were similar to that found at D21, but there was greater within group variability in AA and SH1293 treatment groups. Mean cartilage area in AA rats was significantly lower (*p <* 0.01) than in Ctrl rats (**Figure 8H**), but with increased variability in cartilage area in AA and SH1293 rats, the positive effect of SH1293 on cartilage area found at D21 was lost at D28. At D21, 16.5% of the articular cartilage was preserved in rats that received SH1293, with a modest increase to 17.4% preservation at D28. At D14, sympathetic nerve density negatively correlated with the articular cartilage area (*r =* -0.94, *p <* 0.05).

**Figure 9:**
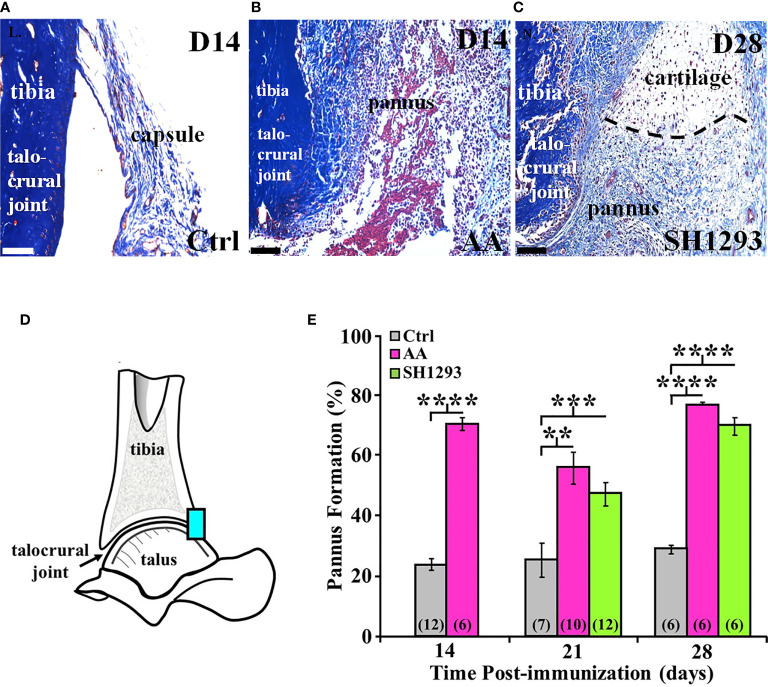
SH1293 Reduced Pannus Formation. In AA and RA, the pannus is fibrovascular or granulation tissue from the hypertrophied synovium with infiltration of macrophage- and fibroblast-like mesenchymal cells that secrete collagen-lytic enzymes. Trichrome-stained tissue of the joints from Ctrl **(A)** or AA and SH1293-treated rats **(B, C),** respectively. Calibration bars = 100 μm. **(A) **At D14 in Ctrl rats, collagen fibers (blue) of fibrous connective tissue form an intact capsule arising from the bone (blue) in the distal tibia; pannus was not present. **(B)** In rats with AA, destruction of the joint capsule and formation of pannus were evident at D14, accompanied by an inflammatory cell infiltration, destruction of the capsule, and accumulation of fibrin (red) and collagenous fibers (blue). **(C)** At D28, in an SH1293-treated rat, an invasive pannus was apparent in the lower half of the image, as well as a pannus-cartilage interface (dashed lines). Pyknotic chondrocytes (dark purple) were present in lacunae in the cartilage. Subchondral bone (deep blue), adjacent to the pannus was eroded (lower left side of the image). **(D)** A diagram of the talocrural joint illustrating the ROI for evaluating cartilage area. **(E)** Morphometric analysis of the pannus. Cartilage area used as a baseline in Ctrl rats did not significantly differ across experimental days of assessment. Pannus formation in AA rats was more extensive at D14, D21 or D28 compared with the percentage of cartilage area established as baseline in Ctrl rats (****p < 0.0001; **p < 0.01; and ****p < 0.0001, respectively). At D21, pannus formation in SH1293 rats was comparable with AA rats, but was greater (***p < 0.001) than Ctrl rats. Similarly, at D28, the percentage of pannus in the arthritic joints from SH1293 rats did not differ from AA rats, but was significantly greater than Ctrl rats (****p < 0.0001). The *n*’s for each group are indicated in parentheses at the base of each bar.

**Figure 9A** shows a representative section of the talocrural joint from a Ctrl rat at D14 with normal subchondral bone and cartilage at the apex of the distal tibial concavity. In AA rats (**Figure 9B**), the development of pannus was evident by D14 and was accompanied by early destruction of the joint capsule. The pannus continued to expand through D28 regardless of treatment (**Figure 9C**). Morphometric analysis at the talocrural joint (**Figure 9D**) revealed significant pannus formation in AA and SH1293 rats by 15.6% and 9.1% at D21 (*p* < 0.01 and *p* < 0.001 compared with Ctrl, respectively) and D28 (*p* < 0.0001), compared with Ctrl rats, respectively. There was no difference between pannus formation between AA and SH1293 treatment groups (**Figure 9E**).

At D21, in AA rats lymphocyte infiltration correlated with pannus formation (*r =* 0.84, *p <* 0.05), and in rats that received SH1293 treatment, bone volume negatively correlated with sympathetic nerve density (*r =* -0.78, *p <* 0.05). At D21, there was also a significant correlation between lymphocyte presence and pannus formation. (*r =* 0.65, *p <* 0.05). At D28, the extensiveness of lymphocytic infiltration inversely correlated (*p <* 0.05) with sympathetic nerve density (*r =* -0.71) in rats treated with SH1293. At D28 significant correlations were observed between pannus formation and bone area (*r =* 0.86, *p <* 0.05) in arthritic rats.

### Reduced Sympathetic Innervation of Arthritic Joints

**Figure 10:**
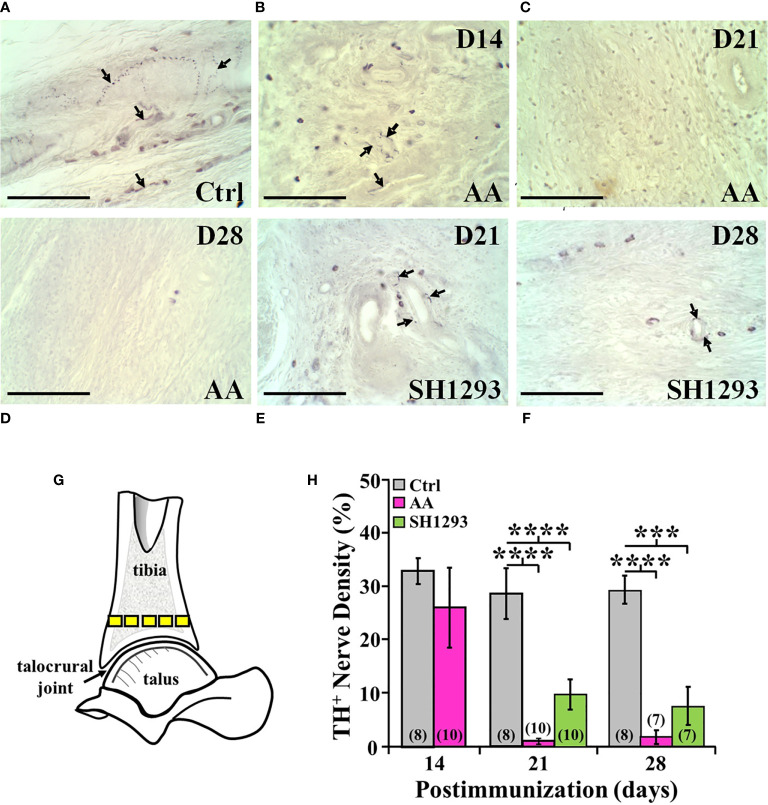
TH^+^ Nerves in the Distal Tibia and Nerve Density Were Reduced at Acute and Severe Disease, and with No Effect of SH1293. **(A)** Representative images from tissue sections from the distal talus immunohistochemically stained from a Ctrl rat demonstrates the presence of TH^+^ nerves (arrows) near the bone/cartilage interface in the trabecular bone. **(B)** Tissue section from an AA rat at D14 demonstrates a decrease in the density of TH^+^ nerves near the bone/cartilage interface in the trabecular bone. **(C)** Image from an AA rat at D21 demonstrates no TH^+^ nerves present near the bone/cartilage interface. **(D)** In this tissue section of the distal tibia, TH^+^ nerves were not present in AA rats at D28. **(E)** Image from an SH1293 rat at D21 demonstrates the sparse presence of TH^+^ nerves around the lumen of an arteriole near the cortical bone. **(F)** An SH1293 rat at D28 continuing to show the presence of TH^+^ cells near the bone/cartilage interface of the trabecular bone. **(G)** Schematic diagram indicating the sample sites for quantifying TH^+^ nerve density in the distal tibia. **(H)** The percentage of the total number of pixels occupied by TH^+^ nerves were quantified morphometrically. At D14, TH^+^ nerve density did not differ in Ctrl and AA rats, although there was greater variability in AA rats. At D21, there was a significant decrease (****p < 0.0001, *n* = 10) in nerve density in AA and SH1293 rats compared with Ctrl rats (*n* = 8). At D28, AA rats had significantly lower (****p < 0.0001, *n* = 7) nerve density than Ctrl rats (*n* = 8). Similarly, nerve density in SH1293 rats was significantly reduced (***p < 0.001, *n* = 7) in nerves compared with Ctrl rats (*n* = 7). The *n’s* for each group at each time point are shown at the base of the bars.

In Ctrl rats, sympathetic nerves entered the cortical bone *via* the nutrient foramen (data not shown). These nerve fibers coursed along the major arteries at multiple sites along the length of the tibia, and extended from these vascular plexuses into periosteum, cortical bone and the medullary space (**Figure 10A**). Few TH^+^ fibers were observed in close proximity to bone cells consistent with norepinephrine spillover from the vascular innervation diffusing across a concentration gradient to bind to β_2_-AR expressed on osteoblast and osteocytes. Activation of these receptors can mediate profound effects on bone turnover. Fourteen days post-disease induction (**Figure 10B**), the abundance of sympathetic nerves in the distal tibia of AA rats was comparable to that observed in the same region of the tibia from Ctrl rats (**Figure 10A**). However, progressive TH^+^ nerve loss was evident between D21 and D28 (**Figures 10C, D**, respectively) compared with Ctrl rats (**Figure 10A**). Quantitation of nerve density in the distal tibia (**Figure 10G**) confirmed a loss of ~60% of TH-immunoreactive nerves in the distal tibia of AA rats by D21 that persisted at D28 (**Figure 10H**; *p* < 0.0001). Much of the distal tibia was devoid of nerves, with occasional nerves observed in bone at D21 and D28 in AA rats (**Figures 10C, D**, respectively, and **Figure 10H**). Compared with Ctrl rats (**Figure 10A**), treatment with SH1293 had a modest sparing effect at D21 (**10E, 10H**) that continued through D28 (**Figures 10A, H**).

## Discussion

This study evaluates the effects of SH1293 on IA in the well-established Lewis rat model. Research, herein extends our previous research demonstrating that SH1293 drives a Th2/Th17-type immune response in secondary immune organs and suppresses systemic inflammation in IA (18, 23). In AA rats, IA presents with a robust systemic and joint-targeted inflammation, consistent with earlier research. Edema, articular leukocyte recruitment, bone and cartilage degradation characterize the affected joints, including the talocrural joint. SH1293 has disease-sparing effects in the arthritic talocrural joint. With treatment started after disease onset, SH1293 significantly reduces soft tissue swelling in the footpads, improves radiographic scores and suppresses joint destruction. These findings are consistent with our previously reported research (18, 23). Its joint sparing effects are due, in part, to suppressed inflammatory cell infiltration, bone sparing by reducing osteoclast numbers (D21/D28), new bone formation, and reduced loss of articular cartilage (D21/D28) compared with AA rats. Disease-sparing effects of SH1293 are most evident during the acute phase of disease (D21), which delayed overall disease progression. SH1293 has no effect on pannus formation or disease-mediated sympathetic nerve loss. These findings are congruent with our research (18, 19, 23) and others (4, 5, 22, 30–32) reporting α- and/or β_2_-AR-targeted anti-arthritic effects on immune function and in skeletal tissues in IA.

In secondary immune organs from rats with IA, we have reported β_2_-AR downstream signal switching from PKA to MAPK/extracellular signal-regulated protein kinase (ERK) pathway in DLNs in rodent models with IA (2, 23). This receptor signaling “switch” promotes production of arthritogenic T cells after disease is established. Here, we find no evidence of aberrant signaling in the talocrural joint based on the well-established actions of α- or β_2_-ARs in skeletal tissues (33–46). Our findings are consistent with SH1293 slowing the disease trajectory in part *via* β_2_-AR signaling mediated *via* cAMP-PKA signal transduction. Existing data supports normal signaling *via* α-AR in IA, and our findings with SH1293 treatment are consistent with normal functioning of α-ARs in the talocrural joint tissues and inflammatory cells (47–49).

### SH1293 Suppressed Inflammatory Cell Infiltration

AA presents with intense migration, extravasation and infiltration of activated immune cells into the talocrural joint. Inflammatory cell recruitment into the joint is evident at D14, and persists and increases at D21 and D28 in AA rats. These activated immune cells organize into cellular nodules. SH1293 significantly reduces cell recruitment during the acute, but not the chronic disease phase. These findings support a transient anti-inflammatory effect of SH1293 that likely contributed to the reduced footpad widths in SH1293 rats. The α-AR antagonist activity of SH1293 may inhibit proinflammatory cytokine production (e.g., tumor necrosis factor (TNF)-α, interleukin (IL)-6, IL-17 and leukocyte extravasation (47–49). This effect may also result from the downregulation of β_2_-ARs in inflammatory cells and high sympathetic tone that is well-established in AA (23, 30). Receptor downregulation effects were further augmented by our drug treatment.

Early in the disease process, SH1293 reduced lymphocyte trafficking into the arthritic joints, consistent with delayed onset of severe IA. In RA patients, Baerwald et al. (50) report an intricate relationship between peripheral blood mononuclear cell reactivity and catecholamine signaling *via* α_1_- and β_2_-ARs that promote RA pathogenesis. In 68-75% of RA patients, clumps of ectopic lymphatic tissue in subchondral bone marrow predicted rapid disease development with 100% accuracy in early disease (50). It was the most significant and sensitive early finding in RA patients, manifesting before synovitis and bone erosions, and increasing risk of bone erosion by six-fold. Our findings of bone sparing and reduced inflammatory cell infiltration during acute IA (D21) is consistent with Baerwald et al. (50), supporting SH1293 as an early treatment strategy to slow disease progression, but perhaps not in late disease as cellular infiltration was similar in SH1293 and AA rats at D28.

### AA Depleted Extra-Articular and Marrow Adipocytes; No Sparing by SH1293

Adipose tissue may contribute to RA pathogenesis (51–58). Marrow adipose tissue was present adjacent to the talocrural joint, and in the inter-trabecular spaces of Ctrl rats. Interestingly, adipose tissue in the bone marrow was not observed in AA rats at any investigated time point after disease onset, regardless of SH1293 treatment. These findings suggest that AA creates an unfavorable microenvironment for bone marrow-derived mesenchymal stem cell (BMSC) differentiation into adipocytes in the talus bone marrow. This hypothesis is supported by Denu and Hematti (51) who report apoptotic cell death in adipocytes exposed to oxidative stress and inflammation, and by Guo et al. (52) who report fat-derived fatty acid-induced apoptosis of preadipocytes in the absence of adipogenic stimuli. Stimulation of β_2_-ARs by SH1293 may promote the loss in marrow adipocytes, by blocking differentiation of preadipocytes into adipocytes (53) or inducing apoptosis (54). Similarly, α-AR antagonist activity of SH1293 may be contributory to adipocyte depletion in AA rats, as phentolamine can suppress DNA synthesis in rat BMSCs (55). The disease and drug-mediated suppression of marrow adipocytes may have negative consequences for bone integrity. These classes of drugs may reduce immunosuppressive cytokine secretion by adipocytes (54), and/or promote influx of Th2 cell-mediating, anti-inflammatory M_2_ macrophages into the arthritic joint (51, 56). Bone marrow dendritic cells exposed to a β_2_-agonist can also promote Th2/Th17 cell expansion, whose activity is also influenced by marrow adipocytes (57). Moreover, the β_2_-antagonist, propranolol can promote trabecular bone formation in mice by regulating adipocyte metabolism (58).

As AA rats do not gain weight, they may model cachectic RA, a less frequent form of disease (59, 60). Higher sympathetic activity drives leptin-mediated depletion of white adipose tissue, cachexia, inflammation and Th2-type immunity (58–60), consistent with cytokine profiles in SH1293 rats. Thus, adipocyte depletion may contribute to the sympathetically mediated anti-inflammatory cytokine profile in AA, and is consistent with bone marrow adipocyte loss with RA progression (59–61). Adiposity in bone marrow negatively correlates with bone mass in mice under inflammatory conditions (61, 62). Adipose-derived stem cells protect periarticular and systemic bone loss in collagen-induced arthritis (CIA) by maintaining trabecular bone structure and suppressing osteoclastogenesis (63). Adipose tissue-derived stem cells also suppress autoimmune T cell responses and increase the percentages of peripheral regulatory T and B cells (63). The sparing effects of SH1293 on trabecular bone may be mediated by indirect effects of β-ARs on adipocytes *via* sympathetic-leptin regulation of body mass to control energy metabolism, as others have reported (64–66). SH1293 may exert some of its effects by targeting adipocytes in early disease. Marrow adipose tissue depletion in the distal tibia may contribute to reduced bone-sparing effect of SH1293 in chronic disease.

### SH1293 Had No Sparing Activity Against Synovitis and Pannus Formation

Pannus formation was similar in all AA treatment groups. In Ctrl rats, the synovium was a monolayer of synoviocytes with no infiltration of inflammatory cells. All rats receiving adjuvant treatment exhibited a thickened synovium indicative of fibroblast-like synoviocyte (FLS) proliferation regardless of SH1293 or vehicle treatment. However, the synovia of SH1293 rats were less infiltrated by inflammatory cells than AA rats. Sympathetic nerves have not been reported in healthy synovial tissue, but synoviocytes express α_2_- and β_2_-ARs (44, 67, 68), indicating the SNS can modulate their functions. Consistent with this, β_2_-agonists exert anti-inflammatory activity in synovial FLS by limiting proliferation and secretion of bone destructive cytokines, TNF-α, IL-1β, and receptor activator of NF-κB ligand (RANKL), and by promoting secretion of osteoprotegerin, the decoy receptor for RANKL that inhibits bone resorption (69). In RA and AA, synoviocytes secrete these inflammatory mediators that drive joint destruction (70–72).

Beta_2_-ARs drive anti-inflammatory activities in FLS (67). Sympathetic nerve loss in AA likely promotes inflammation and uncontrolled proliferation of synoviocytes that accompanies progressive bone and cartilage erosion. Excessive sympathetic activation in AA and RA induces nerve toxicity and subsequent retraction that may reduce sympathetic activation of FLS. Additionally, reduced β_2_-AR signaling in synoviocytes may result from receptor desensitization and down-regulation (67). Based on the reported pathologic β_2_-AR downregulation in synoviocytes in RA, SH1293 may further desensitize/down regulate β_2_-ARs. Lost receptor signaling removes a normally suppressive mechanism of pannus formation and its invasion into adjacent bone. Destruction of sympathetic nerve terminals innervating the synovium and loss of β_2_-AR responsiveness likely explain our findings that SH1293 failed to inhibit hyperproliferation and invasiveness of the pannus. However, SH1293 may have delayed pannus formation and reduced production of pannus-promoting proinflammatory cytokines in AA rats early in the disease. However, it lost efficacy with dampened β_2_-AR signaling capacity in severe disease. Consistent with this interpretation, Wu et al. (67) describe β_2_-AR signaling in synoviocytes from RA patients and in animal models. Reduced G protein-coupled receptor kinase 2 (GRK2)-mediated β_2_-AR signaling by receptor desensitization and/or increased receptor downregulation may sabotage the normal protective β_2_-agonist effect. Thus, PKA/GRK2- or MAPK/ERK-targeted interventions may provide a better strategy for reining in aggressive FLS in RA. Moreover, this treatment strategy may suppress abnormal GRK-2-mediated phosphorylation of β_2_-AR in splenic lymphocytes in IA (23), RA and possibly other inflammatory diseases (67, 68).

### SH1293 Transiently Spared Subchondral Bone but Spared Trabecular Bone Through D28

SH1293 significantly prevented the loss of bone area at acute and chronic disease. Morphometric analysis of bone revealed interesting differences in bone-sparing effects of SH1293 in subchondral and trabecular bone. Bone-sparing effects were most striking when compared with the significant loss of bone area of AA rats. In trabecular bone, SH1293^hi^ rats reduced the loss of bone area at D21 and D28. The sparing effects of SH1293 on bone area persisted through D28. Our findings support re-established homeostatic osteoblast-osteoclast balance at a new lower set point in acute and chronic disease. Still, bone area did not change in five SH1293^lo^-responder rats.

In most of the SH1293 rats, microCT scans revealed less bone resorption at the talocrural joint, and in other small bones of the foot at severe disease not captured by bone volume quantitation. A decline in bone volume was evident in AA rats at D14, continued to decline by acute disease, but then stabilized through severe disease. AA reduced subchondral bone volume by ~20% and ~50% at D21 and D28, respectively, compared with Ctrl rats. At D28, bone volume was ~13% and 75% higher in SH1293^hi^ rats than in Ctrl and AA rats, respectively. SH1293 spared subchondral bone in the distal tibia at D21, with no significant difference found between SH1293 and Ctrl rats. However, as IA progressed to severe disease (D28), the sparing effect of SH1293 on bone volume was lost.

Variability in bone integrity across animals was most evident for the bone area, possibly due to larger sampling size and/or the type of bone (trabecular) targeted for analyses. TRAP-stained trabecular bone revealed a bimodal effect of SH1293. A non-significant trend towards lower bone area was found in AA rats at D14, which may represent a small loss in trabecular bone as osteoclast numbers were higher in AA than in Ctrl rats at this time point. Moreover, there was greater within-group variability in bone area in Ctrl and AA rats at D14, and AA rats at D28. In AA rats, bone area was slightly, but not significantly, lower at D14 than in Ctrl rats. Severe reduction in bone area is evident in AA rats at acute and severe disease. SH1293 reduced trabecular bone loss in more than half of the AA rats at acute (D21) and severe (D28) disease. Bone area was similar in SH1293^lo^ and AA rats, and inversely related to osteoclast number in trabecular bone. Osteoclasts were significantly more abundant in AA and SH1293^lo^ responders than in SH1293^hi^ and Ctrl rats at all three time points. Collectively, these findings confirm our previous research showing bone-sparing effects of SH1293 by X-ray analyses (19). These findings indicate that stimulating β_2_-ARs concurrently with α-AR inhibition can markedly slow AA-induced trabecular bone loss in about two-thirds of the rats treated with SH1293. Notably, the bone area was reduced in SH1293^lo^ rats at D21 and D28, an effect that was consistent with higher mean osteoclast numbers in these rats. The shift towards greater osteclastogenesis may be attributed to greater differentiation of mesenchymal stem cells toward osteclastogenesis (63). In AA and SH1293^lo^ rats, an increase in osteoclasts in trabecular bone suggests greater bone turnover in SH1293^lo^ rats in chronic disease. In contrast, comparable osteoclast numbers in Ctrl and SH1293^hi^ rats support a beneficial role in SH1293^hi^-responder rats. The reason for the high and low responses in SH1293 rats is not clear, but home cage housing of dominant vs. subordinate rats may be contributory. Alternatively, SH1293 may indirectly exert bone-sparing effects by shifting disease-inducing Th1/Th17 vs. Th2 cell balance, as we have reported in the AA rat model, and Straub et al. have reported in the CIA mouse model of RA (22). The role of hormones secreted by the hypothalamic-pituitary-adrenal cortical axis and/or the parasympathetic nervous system that regulate the Th cell function could also be contributory (4, 8, 34).

SNS regulation of bone remodeling is complex, but it is clear that both β_2_- and α_1B_-ARs are expressed in and regulate osteoblast/osteoclast remodeling of bone. Stimulation of α_1B_-ARs and β-ARs expressed in bone are well documented to exert opposing actions on bone volume. In osteoblasts, our data suggest a similar mechanism may explain the bone-sparing effects of SH1293. This hypothesis is proposed based on findings that contradict the expected normally suppressive β_2_-AR effects of ligand binding in bone cells. Under physiologic conditions, activation of β_2_-ARs and inhibition of α_1B_-AR expressed in osteoblasts suppress osteoblastogenesis and augments osteoclastogenesis to drive bone resorption, respectively (72, 73). Thus, early bone-sparing of SH1293 in the acute phase of disease cannot be explained by increased β_2_-AR stimulation in osteoblasts. Instead, the observed loss of adipocytes may provide an alternative mechanism for the early bone-sparing and subsequent decline in the effect of SH1293 on bone as SH1293-targeted adipocytes are depleted.

Bone loss was most severe in trabecular bone and was accompanied by the loss of adipocytes. The loss of adipocytes in the bone marrow may be explained by a shift from adipogenesis toward osteoblastogenesis to meet the increased need for bone repair and bone sparing through D21 (74), and BMSC exhaustion at D28 (75, 76). BMSC exhaustion would affect bone integrity and therefore may be an important determinant in bone degradation in AA by reducing energy resources to support osteoblast function, and shifting differentiation of bone marrow stem cells toward osteoblastogenesis and subsequent BMSC exhaustion.

Direct inhibitory actions of SH1293 on α-ARs suppress bone formation, and may explain suppressed abnormal bone formation in the talocrural joint observed in this study. Stimulation of α_1_-AR signaling is important in up-regulating osteoblast functions that are necessary for bone formation, turnover and maintenance (39, 43, 77). Norepinephrine binding to α_1B_-ARs expressed on cultured osteoblasts increases osteoblastogenesis by Gi/o-coupling that block K^+^ channels in osteoblasts (73, 78). Signaling *via* α_1B_-AR is reported to regulate bone mass by regulating CCAAT/enhancer-binding protein delta (Cebpd), a protein important in genes that regulate immune and inflammatory responses. In bone, Cebpd-mediated transcription of insulin-like growth factor-1 stimulates osteoblast replication and differentiation (40). However, mice with chronic sympathetic hyperactivity display high bone mass with increased bone formation and reduced bone resorption (78). If similar effects observed in knockout mice occur with α-AR blockade in AA-induced SNS hyperactivity, α-AR antagonism by SH1293 treatment may contribute to bone sparing in the talocrural joint through D21.

The early bone-sparing effects of SH1293 may be explained by sympathetic regulation of locally-released leptin. First, α- and β_2_-AR activation augments and suppresses adipocyte-secreted leptin, respectively (53–66). Leptin’s control over osteoblast function is an important regulator of energy metabolism through direct and indirect influences on bone integrity in IA (54–63). Leptin acts on osteoblasts to suppress their proliferation, drive bone mass accrual, and regulate osteoblast survival once they’ve matured from precursor BMSCs (58, 59). Additionally, leptin modulates insulin sensitivity by reducing osteocalcin bioactivity in osteoblasts. Leptin secretion is blocked by adiponectin and adipsin, which are major regulators of bone mass and energy metabolism, in part by reducing local SNS activity necessary to increase bone formation and reduce energy expenditure (54, 55, 61, 66). Thus, these fat-derived mediators regulate two opposing mechanisms, bone mass accrual and bone destruction by blocking adipocyte-secreted leptin, important regulators of energy metabolism (58, 59, 63). Collectively, this literature supports the notion that energy balance skews toward anabolic metabolism at the expense of bone mass accrual in IA, consistent with arthritis-induced cachexia. SH1293 may counteract the shift toward catabolic energy metabolism *via* its action on α- and β_2_-ARs, which indirectly contributes to its bone sparing effects in the talocrural joint. This hypothesis is further supported by research showing that the SNS-leptin pathway regulates osteocalcin and insulin (66), which in turn raise each other’s activities/secretion as a feedforward loop and also promotes bone formation.

### SH1293 Influence on Subchondral Bone, Articular Cartilage and Chondrocytes

In non-AA rats, sympathetic nerves supplied the periosteum, subchondral plate and underlying trabecular bone of the distal tibia. In AA rats, these nerves were absent by D21, where there was extensive bone loss proximally and distally to the talus. These observations are consistent with a bidirectional attack in bone (79) that promotes nerve retraction. Treatment with SH1293 provided a significant benefit for preserving cartilage at D21 that continued through D28, along with greater presence of TH^+^ nerves along the vasculature. Both α- and β-ARs are abundantly expressed in chondrocytes (80) and regulate cartilage turnover (80, 81), but the cartilage-sparing effects mediated by SH1293 are consistent with a primary effect of α_2_-AR antagonism (80, 81). Alpha_2A_-ARs are expressed in condylar cartilage cells and promote degenerative cartilage remodeling by increased expression of metalloproteases-3 and -13, and RANKL (81). SH1293 inhibition of α_2A_-AR is consistent with this mechanism of cartilage sparing. In contrast, β_2_-AR stimulating effects of SH1293 (i.e., terbutaline) have previously been demonstrated to promote chondroclastic activity by inducing RANKL (45, 82). At high, but not low concentrations, norepinephrine can inhibit chondrogenesis in BMSCs *via* ERK1/2 and PKA mediation, effects that are blocked by the β_2_-AR blocker, propranolol (83). Our data indicates that treatment with SH1293 delayed and suppressed articular cartilage resorption in the distal tibia in arthritic rats, findings that support direct inhibitory effects of terbutaline on chondrocyte proliferation. Articular cartilage sparing at the talocrural joint of SH1293-treated rats was marked in that it was similar to non-arthritic Ctrl rats, supporting that SH1293 preserves cartilage in arthritic rats.

### SH1293 Did Not Prevent Arthritis-Induced Sympathetic Nerve Loss

TH^+^ sympathetic nerve fibers were present in the synovial tissue and subchondral bone marrow in non-AA rats, but were lost in AA rats with no discernible sparing effects of SH1293. These findings are consistent with qualitative reports of nerve retraction within the distal joints in other models of IA, such as CIA in mice (9) and other models of inflammation (84, 85). Moreover, dying back of sympathetic nerves is consistent with chronically high firing rates in sympathetic nerves that can cause oxidative nerve damage. Research by Straub et al. further supports an increased expression of the nerve repellent, neuropilin-2 (86) that may be responsible, at least in part, for the sympathetic nerve loss in the distal tibia. An inverse relationship exists between the loss of nerves in arthritic bones and the induction of bone production by norepinephrine (58, 85).

Dysfunction of sympathetic neurotransmission is an early pathologic event in RA and in rodent models of RA (2, 17, 18, 23, 87–92). Consistent with these reports, the densities of sympathetic nerves in the synovium are similar in control and AA rats shortly after disease onset, but dramatically reduced at acute and chronic disease. Sympathetic nerve loss in synovial tissue of patients with RA (90) is associated with increased sympathetic activity (45), and oxidative stress increases their vulnerability to injury (87). In the AA model, sympathetic nerves sprout into the upper dermis of the skin (87), where cutaneous sensory nerves contribute to chronic skin inflammation and cutaneous pain in IA (87). Straub et al. (88) found greater sympathetic nerve density in metabolically activated adipose tissue surrounding human synovium, and murine DLNs in CIA mice. Resident cells in bone and articular tissues express α- and β-ARs that respond to catecholamine neurotransmitters released from sympathetic nerves. In bone, β_1/2_-AR activation promotes bone loss, but competitive blockade with propranolol had a small, but significant effect on bone, specifically increasing trabecular number and reducing trabecular spacing (58). The β_2_-AR-mediated actions of SH1293 on multiple targets in bone (i.e., osteoblast, osteoclasts, adipocytes, fibroblasts, inflammatory infiltrates) may have direct and indirect actions with opposing actions on bone metabolism occurring across disease progression. Consistent with indirect β_2_-AR actions on skeletal tissues, Li et al. (89) found protective effects of the β-blocker, nebivolol against IL-1β-induced type II collagen destruction mediated by matrix metalloproteinase-13, whereas direct effects of β_2_-ARs on fibroblasts are anti-fibrotic.

In contrast to our finding, another study by Longo et al. (87) reports sympathetic, but not sensory, nerve sprouting in inflamed joints and adjacent skin four weeks after CFA treatment administered directly into the hind paw. Partially consistent with this report, other researchers (93–96) found that both sensory and sympathetic nerves sprout into the arthritic knee joint in geriatric and young mice, after this joint was injected with CFA. Differences in sprouting of sensory nerves between the two studies may reflect the differences in timing of the CFA injections and/or the site of CFA injection (i.e., the dermis of hind paw vs. the base of the tail). Collectively, these findings suggest the site of inoculation to induce arthritis or timing of CFA injection may influence the density and distribution of sympathetic nerves in the hind limb, such that immunization into the hind paw promotes sprouting of sympathetic nerves into that site, but not immunization of CFA into the base of the tail, as in our study. Additionally, inflammation, altered trophic support, and/or autoantibodies against ganglionic neurotransmitters (97–100) may influence AR expression and/or function in the arthritic joint, and therefore the functional activity of SH1293.

In non-AA rats, noradrenergic nerves innervated the vasculature and tissue components in the synovium, trabecular and subchondral bone, periosteum, and fat tissue in the marrow cavity, and surrounding the synovium (88, 93). At D21 and D28, AA reduced the density of TH^+^ nerves in these compartments of the distal tibia. It is well established that oxidative free radicals associated with inflammation promote sympathetic nerve loss. Moreover, nerve repellants, like semaphorin 3F, are produced in arthritic joints that influence sympathetic nerve density (101). Fewer nerves in arthritic bones may deplete an important extracellular source of norepinephrine for norepinephrine transporter-mediated uptake into osteoblasts necessary for bone accrual and remodeling (65, 102, 103). Activation of β-ARs in BMSCs can inhibit osteoblast anti-adipogenic potential (102, 103). Garimella et al. (63) reported that adipose-derived mesenchymal stem cells can prevent inflammation-induced bone loss in the murine CIA model by reducing osteoclast precursors and promoting immune tolerance. If a similar mechanism is found in AA, norepinephrine uptake into osteocytes may buffer the bone catabolic actions of norepinephrine that is released by sympathetic nerves by reducing norepinephrine available to bind β-AR receptors expressed on osteoblasts.

At both acute and chronic phases of IA, SH1293^hi^ and SH1293^lo^ responders were identified based on osteoclast number in talar trabecular bone from arthritic rats. The reason for this dichotomy is not clear. Differences in α_2A_-AR mRNA stability in osteoblasts may contribute to differences in bone remodeling and bone loss (77, 104). Moreover, sensory or parasympathetic nerves may also be contributory, as they are involved in the pathology of AA and RA (4, 34, 105). Severity of the inflammatory response and/or skewing of the inflammatory response in diverging directions may also contribute to the dichotomy in response to SH1293 treatment. Inflammation may induce bone marrow edema in IA, which is associated with greater osteoclast number in these lesioned sites (105). Another variable that we did not track was dominant versus subordinate rats in each home cage, which may, in combination with disease severity, contribute to divergent findings (106). Disease manifestations may be sensitive to the housing environment, as stress modulates the immune system. A transcriptional study of murine osteoclast differentiation reported findings that may explain our dual response, based on highly time-dependent waves of distinct expression of signaling and receptor molecules that dynamically regulate bone physiology (107).

### Summary and Conclusions

Our strategy in this study was based on our previous research identifying a promiscuous switch in β_2_-AR signal transduction. The switch in signal transduction by β_2_-ARs from PKA to ERK1/2 in DLNs occurs between disease induction and the chronic phase of AA. This switch in β_2_-AR signaling is expected to drive the production of arthritogenic T cells in DLNs and promotes a proinflammatory disease state. In contrast, β_2_-AR signaling in the spleen was shut off due to receptor down-regulation (decreased receptor numbers) and desensitization (no signaling). This may be an important protective mechanism to suppress systemic inflammation. It remains unclear whether promiscuity in β_2_-ARs in AA is confined to immune cells or extends to AR signaling in affected joint tissue, but understanding the extent to which signal switching or dual signaling cascade activation, as well as “when” and “where” promiscuous β_2_-AR signaling occurs in important disease-mediating targets is critical for understanding the pathophysiology of RA. Our data may shed at least some light on this question. Specifically, we assessed the histological effects of SH1293 on specific types of tissue in the hind limb, which is well-documented to express β_2_- and α-AR. The effects of SH1293 were assessed during the effector phases of disease on arthritis severity and the pathology of the talocrural joint in the rats with AA. We report that SH1293 reduced soft tissue swelling by suppressing inflammation and spared bone by reducing osteoclast numbers and cartilage loss during acute disease. The directional effects of SH1293 reported herein on the arthritic distal tibia and talocrural joint are consistent with conventional β_2_-AR signaling *via* cAMP. There was no effect of SH1293 on pannus formation or protection against sympathetic nerve loss in the arthritic joints. Our findings predominantly support a transient, but significant disease-sparing effect of SH1293 in AA during the acute disease phase that provides insight for improving treatment strategies in RA, particularly during early in disease.

## Data Availability Statement

The raw data supporting the conclusions of this article will be made available by the authors, without undue reservation.

## Ethics Statement

The animal study was reviewed and approved by Animal Use and Care Committee, at the Hoover Arthritis Research Center, Banner Health Research Institute in Sun City, AZ. All animals were treated in accordance with the approved Animal Use and Care protocol.

## Author Contributions

DB: conception and study design, conducted experiments, data acquisition, data analysis, manuscript review and discussion, and wrote the manuscript. CW: conception and study design, conducted experiments, data acquisition, data analysis, and wrote the manuscript. JW: contributed to experimental design, expertise in joint histology, data analysis, and manuscript review. DL: conception and study design, conducted experiments, data acquisition, data analysis, discussion of results, and wrote the manuscript. All authors contributed to the article and approved the submitted version.

## Funding

This study was supported by R01 NS055673 and an intramural grant from the Department of Pathology and Human Anatomy.

## Conflict of Interest

The authors declare that the research was conducted in the absence of any commercial or financial relationships that could be construed as a potential conflict of interest.

## Abbreviations

AA, adjuvant-induced arthritis; ANOVA, analysis of variance; ARs, adrenergic receptors; BMSC, bone marrow-derived mesenchymal stem cell; Cebpd, CCAAT/enhancer-binding protein delta; CIA, collagen-induced arthritis; CFA, complete Freund’s adjuvant; Ctrl, non-treated non-arthritic controls; D, day; DLNs, hind limb draining lymph nodes; ERK, extracellular signal-regulated protein kinase; FLS, fibroblast-like synoviocytes; GRK2, G protein-coupled receptor kinase 2; H&E, hematoxylin and eosin; IA, inflammatory arthritis; IL, interleukin; MAPK, mitogen-activated protein kinase; MSC, mesenchymal stem cell; micro-CT, micro-computed tomography; PBS, phosphate-buffered saline; PKA, protein kinase A; RA, rheumatoid arthritis; RANKL, receptor activator of NF-κB ligand; ROI, region of interest; SH1293, adjuvant-induced arthritic rats treated with SH1293; SNS, sympathetic nervous system; SEM, standard error of the mean; TH, tyrosine hydroxylase; TNF, tumor necrosis factor; TRAP, tartrate-resistant acid phosphatase.
